# Network Rewiring in Cancer: Applications to Melanoma Cell Lines and the Cancer Genome Atlas Patients

**DOI:** 10.3389/fgene.2018.00228

**Published:** 2018-07-10

**Authors:** Kuan-Fu Ding, Darren Finlay, Hongwei Yin, William P. D. Hendricks, Chris Sereduk, Jeffrey Kiefer, Aleksandar Sekulic, Patricia M. LoRusso, Kristiina Vuori, Jeffrey M. Trent, Nicholas J. Schork

**Affiliations:** ^1^J. Craig Venter Institute, La Jolla, CA, United States; ^2^Department of Bioengineering, University of California, San Diego, San Diego, CA, United States; ^3^Sanford Burnham Prebys Medical Discovery Institute, La Jolla, CA, United States; ^4^The Translational Genomics Research Institute, Phoenix, AZ, United States; ^5^Department of Medical Oncology, Yale Cancer Center, Yale University, New Haven, CT, United States; ^6^Department of Psychiatry, University of California, San Diego, San Diego, CA, United States

**Keywords:** network rewiring, melanoma, pathway analysis, bioinformatics and computational biology, machine learning, drug interactions, simulation models, data science

## Abstract

Genes do not work in isolation, but rather as part of networks that have many feedback and redundancy mechanisms. Studying the properties of genetic networks and how individual genes contribute to overall network functions can provide insight into genetically-mediated disease processes. Most analytical techniques assume a network topology based on normal state networks. However, gene perturbations often lead to the rewiring of relevant networks and impact relationships among other genes. We apply a suite of analysis methodologies to assess the degree of transcriptional network rewiring observed in different sets of melanoma cell lines using whole genome gene expression microarray profiles. We assess evidence for network rewiring in melanoma patient tumor samples using RNA-sequence data available from The Cancer Genome Atlas. We make a distinction between “unsupervised” and “supervised” network-based methods and contrast their use in identifying consistent differences in networks between subsets of cell lines and tumor samples. We find that different genes play more central roles within subsets of genes within a broader network and hence are likely to be better drug targets in a disease state. Ultimately, we argue that our results have important implications for understanding the molecular pathology of melanoma as well as the choice of treatments to combat that pathology.

## Introduction

Many studies leveraging genomic assays explore associations between genes and diseases. If any associations are found then strategies to make broader claims about genetically-mediated processes, networks, and pathways that influence those diseases are pursued; for example, by studying the expression levels or protein function of disease-associated genes. Identifying genetically-mediated molecular physiologic processes underlying a disease could lead to insights into how to combat or treat the disease. One strategy for identifying molecular physiologic processes responsible for a disease is to measure gene expression levels in diseased and non-diseased tissues. Differentially expressed genes could then be extracted to determine if all, or some subset, of those genes participate in a coherent network or contribute to particular processes affecting disease pathogenesis. Although intuitive, gene expression analyses like this are complicated. For example, one strategy for making claims about whether or not the differentially expressed genes (DEGs) all participate in a particular network is to compare a list of DEGs to databases, such as Kyoto Encyclopedia of Genes and Genomes (Kanehisa and Goto, 2000) (KEGG) or WikiPathways (Pico et al., 2008; Kelder et al., 2012; Kutmon et al., 2016), which provide network, function, or pathway information associated with each gene. If the DEGs match lists of genes in, e.g., known networks, then it can be inferred that those networks are likely to be contributing to the pathogenesis of the disease (Costanzo et al., 2010; Blomen et al., 2015).

Such strategies do not address crucial questions about the relationships between the genes, i.e., their correlations, which might be necessary to determine the potential effect of a drug on a network of genes, however defined. For example, it might be necessary to know if the inhibition of one gene in the network will lead to the inhibition (or excitation) of another. In order to assess the relationships between the genes, and whether those relationships are themselves different in the diseased or normal state, one could pursue either a “bottom-up” approach or “top-down” approach. In the bottom-up approach, a single gene is taken as central and a network of associated genes, based on their correlations with the central gene, is constructed. This is done in the context of both the diseased state and non-diseased state and differences between the networks are assessed (Gorban et al., 2010; Censi et al., 2011). In order to assess these differences one could take advantage of a number of statistical methods such as those associated with the analysis of complex network invariants (Chang et al., 2013). In the top-down approach, a basal “wiring” diagram or topology describing connections between genes in the network is exploited. The genes whose expression levels are assessed are overlaid on this network to see which genes, or correlation levels between genes, appear to be disrupted in the context of this network. Unfortunately, most network topologies or wiring diagrams have been devised from studying genes in the normal (i.e., non-diseased) state (Califano, 2011; Ideker and Krogan, 2012; Snider et al., 2015). This is problematic if the networks have become “rewired” (i.e., the topology or connections between the genes is fundamentally different) in the disease state, since important differences between the networks would not be captured by having the expression levels of the genes overlaid over a network that simply doesn't apply in the disease state. That is, differences at the more holistic or broader network level, and the choice of which specific genes to explore in further studies that is guided by the network topology obtained in the normal state may not point out crucial relationships unique to the disease state.

**Figure 1:**
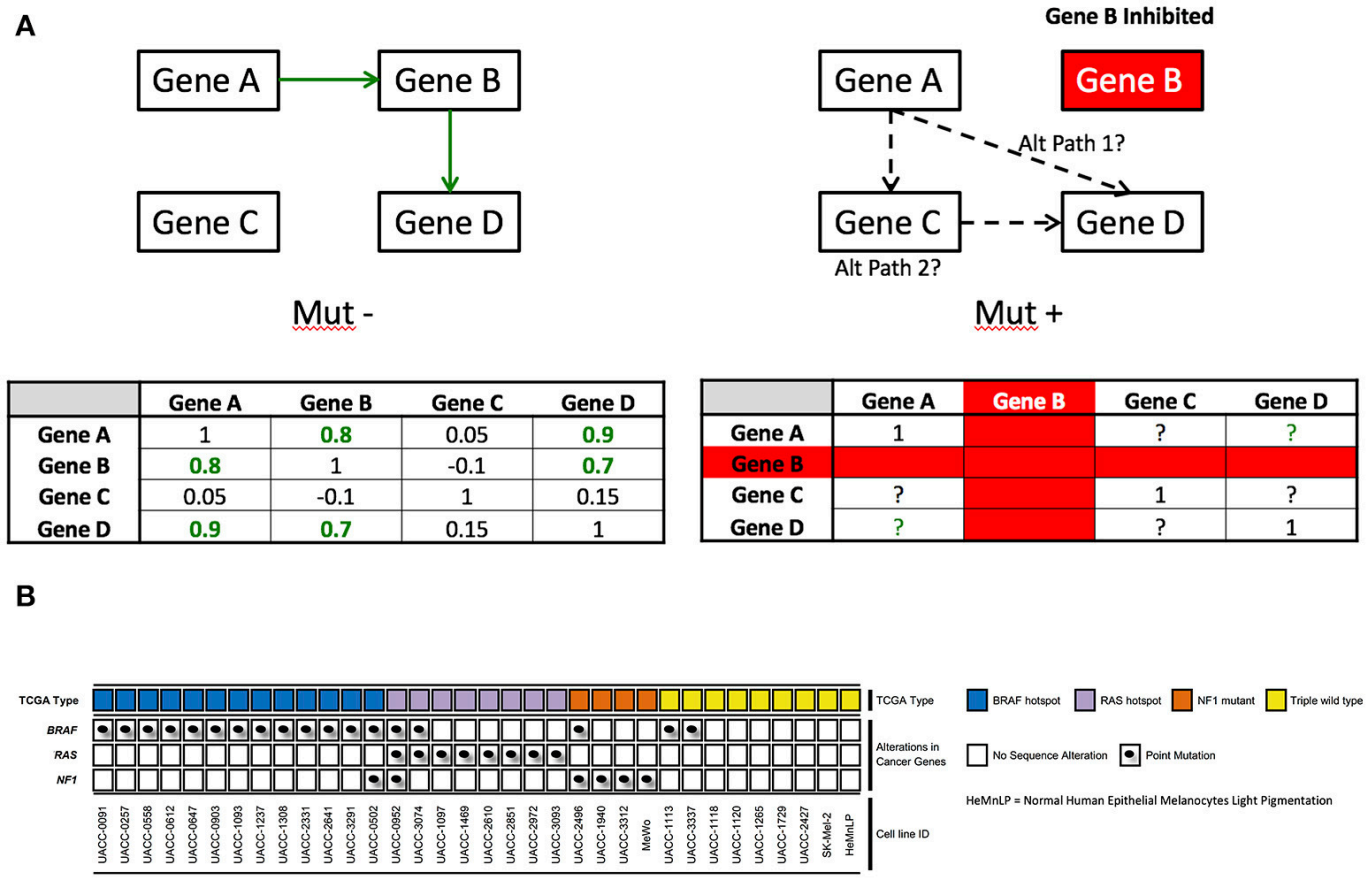
**(A)** Example of network rewiring that could occur. Under ideal conditions, Gene A is co-regulated with Gene D through gene B. When gene B is inhibited, there could be a compensatory mechanism for which gene A communicates with gene D–in this example, that is potentially gene C. **(B)** Mutation status of 33 melanoma cell lines and HeMnLP processed with whole genome microarray gene chips at the Translational Genomics Research Institute. TCGA type calls are based on known pathogenic hotspots for BRAF and RAS (e.g., BRAF V600 mutations), but are more open-ended for NF1. Point mutations are provided for BRAF, RAS, and NF1. Cell lines are ordered by TCGA mutation type, point mutation, and then cell line number.

As an example of this problem, consider the fact that many drug target identification and modulation studies focus on how drugs impact levels of gene expression or protein expression in wild-type, normal, or conveniently available, cell lines (St Onge et al., 2007). If the information flow and connections between genes are fundamentally different in the diseased state, then extrapolating a drug's effects on genes or protein levels in the normal state to that of the diseased state may be problematic (see Figure 1A for simple hypothetical example). Another issue with the characterization of the relationships between genes is that there may be many genes in a given network, creating a large number of relationships to consider (Hofree et al., 2013). This creates computational and inferential challenges. In addition, such analyses require that one pre-specify the groups of genes whose pairwise gene relationships are to be contrasted (e.g., BRAF mutant positive tumors vs. BRAF wild-type tumors in melanoma) and choosing those groups may reveal connections very specific to those groups, which may be limiting if broader connections between genes are of interest.

To identify differences in network architecture within a given data set, one could pursue a “supervised” or “unsupervised” approach. Supervised approaches, such as Evaluation of Dependency DifferentialitY (Jung and Kim, 2014) (EDDY) analysis, Gene Set Enrichment Analysis (Mootha et al., 2003; Subramanian et al., 2005) (GSEA), and Gene Set Co-expression Analysis (Choi and Kendziorski, 2009) (GSCA), focus on groups defined *a priori*, and as a result are limited to the group definitions. Unsupervised methods do not require that the groups be specified *a priori*, but rather take all the, e.g., cells, under study and seek to identify groups that may or may not recapitulate groups defined by known factors, such as the presence of mutations, and therefore often generate results that might be difficult to interpret or reconcile with the results of other unsupervised methods (Getz et al., 2000). In addition, most available unsupervised genetic analysis methods focus on exploring differences in the means, or mean vectors, of the factors being studied within and among groups (e.g., gene expression values, protein abundances, etc.) as a way of determining which factors may be contributing to differences between them. However, if, as noted, the rewiring of the genetic networks among the groups upsets the relationships or correlations between the genes (i.e., and not the just mean or average differences between them), then focusing on mean gene expression levels will not identify the group differences, although this effect might be resolved using various methods that allow for an understanding of identifiable sources of variation in expression levels, such principal components (Censi et al., 2011).

We consider the value of both supervised and unsupervised approaches in the analysis of network data (Chang et al., 2013; Yang et al., 2014; Klein et al., 2016). We assessed evidence for network rewiring in a variety of melanoma drug screen data as well as data from The Cancer Genome Atlas (Weinstein et al., 2013) (TCGA) tumor profile data sets. We further consider the implications such rewiring might have for making claims about cell line responses to drugs. Specifically, we pursued supervised analyses of genetic networks by stratifying melanoma cell lines into groups defined by BRAF and/or KRAS mutation status and then evaluating differences in the overall network structure (i.e., relationships between genes). Using this strategy, we sought to identify genes that appeared to have a more central role in those networks. We then performed network analyses with patient samples from TCGA melanoma data. We observed that the cell lines exhibited a greater degree of network rewiring or differences than the patient samples and therefore leveraged unsupervised methods to explore the variation in network behavior. These analyses allowed us to identify potential drug targets. In addition, we also show that these analyses could shed light on how to match patient genetic profiles to specific treatments by exploiting the Drug Gene Interaction database (Wagner et al., 2016) (DGIdb). Ultimately, our proposed methods provide a strategy for identifying unique network features among the expression levels of genes in tumors and how one could exploit those unique features for drug targeting purposes.

## Results

### Unsupervised multivariate mixture model analysis of the transcriptomes of melanoma cell lines

**Figure 2:**
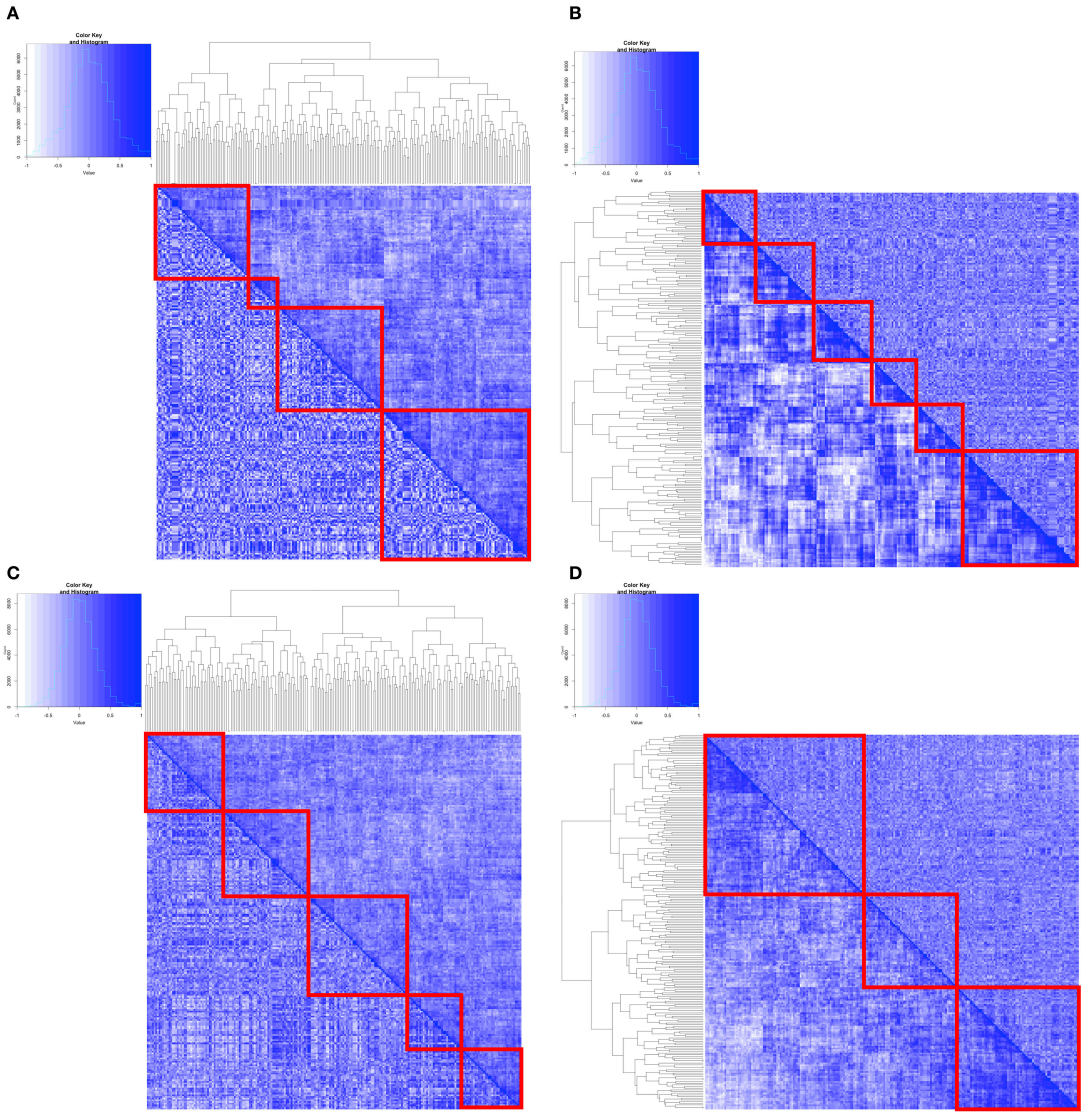
Pairwise correlation between MAPK Pathway genes from the identified clusters using flexmix were used to generate heatmaps. Upper right hand half of matrix were correlation coefficients from cluster 1, whereas lower left hand half of matrix were correlation coefficients from cluster 2. Hierarchical clustering was subsequently performed using either distances from cluster 1 coefficients **(A,C)** or cluster 2 coefficients **(B,D)**. **(A,B)** are from flexmix derived clusters on SU2C melanoma cell lines **(C,D)** are flexmix derived clusters on CCLE melanoma cell lines.

As noted in the Methods section, we extracted genes from the WikiPathway version of the MAPK pathway (*n* = 224). These 224 genes were then mapped to the probesets used in the Affymetrix gene expression chips applied to the CCLE and TGen cell lines at our disposal for these analyses based on the maximum average intensity for each gene (Supplemental Table 2). We also obtained gene expression levels for these genes from the RNA-seq data available on the TCGA melanoma samples. However, only 216 or these 224 genes could be confidently mapped to the TCGA RNAseq data. We applied mixture model analysis as implemented in the flexmix R module (see Materials and Methods) to identify gene expression-based clusters in the SU2C melanoma cell lines. Across the different datasets, two groups of cell lines consistently emerged. For the SU2C cell lines the two groups emerging from this analysis were made up of 27 and 7 cell lines. In applying it to the CCLE melanoma cell line group sizes of 39 and 19 were identified (Table 1). Moreover, for the TCGA data, the two groups were made up of 401 and 72 tumors. When all the CCLE cell lines, including the melanoma cell lines, were considered together the groups emerging had 375 and 542 cell lines. Density plots for the posterior probability of the flexmix-identified groups revealed that the clusters were most distinct in the SU2C melanoma cell lines (Figures 2A,B). This is also evident when viewing the number of edges present in comparing graphs with identical nodes (Supplemental Figures 3, 4). Interestingly, when we used permutation tests to assess the statistical significance of the mean gene expression levels between the two groups we found that mean equality tests did not reveal statistically significant differences. However, tests of the equality of the correlation matrices between the two groups yielded statistically significant *p*-values for the TCGA melanoma patients and for the CCLE cell lines when all cell lines were considered, and moderately significant *p*-values for only the CCLE melanoma cell lines were considered, but a non-significant *p*-value for the SU2C melanoma cell lines. However, we found that in each cohort, the groups identified from the mixture model analyses had statistically significant differences in expression profiles based on at least one statistic of the omnibus statistics used (i.e., either the LLR, delta AIC, or delta BIC statistics; see Table 1).

**Table 1:** Comparing a priori called clusters within SU2C melanoma cell lines (MCL), CCLE MCL, CCLE all cell lines (ACL), and TCGA melanoma patients.

	**SU2C MCL**	**CCLE MCL**	**CCLE ACL**	**TCGA Mel**
**MEAN EQUALITY TEST**				
T-Test Statistic	4.99	6.14	6.60	4.31
df	223.00	223.00	223.00	216.00
*p*-value	1.23E−06	3.66E−09	3.03E−10	2.44E−05
Simulation p-value	3.09E−01	4.33E−01	3.45E−01	3.58E−01
**CORRELATION EQUALITY TEST**				
Chi-sq Statistic	1320.27	1915.88	66408.10	76275.79
df	561.00	1653.00	22791.00	22366.00
*p*-value	4.30E−63	6.50E−06	0.00E+00	0.00E+00
Simulation *p*-value	8.89E−02	4.60E−02	9.99E−04	9.99E−04
**FLEXMIX 2 GROUPS VS. 1 GROUP**				
LLR Statistic	9.35	0.44	9.48	34.40
LLR Permuted *P*-Value	8.99E−03	3.79E−01	1.30E−02	9.99E−04
Delta AIC Statistic	−3.35	5.56	−3.48	−28.40
Delta AIC Permuted *P*-Value	8.99E−03	3.80E−01	1.30E−02	9.99E−04
Delta BIC Statistic	1.23	11.74	10.98	−15.92
Delta BIC Permuted *P*-Value	9.99E−04	2.90E−02	3.20E−02	9.99E−04
**FLEXMIX 3 GROUPS VS. 1 GROUP**				
LLR Statistic	9.35	0.51	9.56	39.45
LLR Permuted *P*-Value	2.80E−02	4.83E−01	3.00E−02	9.99E−04
Delta AIC Statistic	2.65	11.49	2.44	−27.45
Delta AIC Permuted *P*-Value	2.80E−02	4.83E−01	3.00E−02	9.99E−04
Delta BIC Statistic	11.80	23.85	31.37	−2.50
Delta BIC Permuted *P*-Value	9.99E−04	9.99E−03	1.05E−01	9.99E−04

*Performed mean vector and covariance equality tests within groups and used log-likelihood ratio permutation-based tests to evaluate strength of one, two, or three groups*.

In order to visualize the relationships between the genes based on their expression levels, and assess their consistency across the two groups identified in each data set, we extracted pairwise spearman correlation coefficients from the correlation matrices and then generated heatmaps associated with those matrices in which the entries above the diagonal were from one group set and those below the diagonal were from the other (Figures 2C,D, Supplemental Figures 5,6). If the correlations were the same the heatmap matrices would be completely symmetric. Clearly, however, the heatmaps suggest differences between the flexmix analysis-based clusters in each of the data sets, although the differences were more pronounced in the SU2C melanoma cell lines and the CCLE melanoma cell lines.

### Does mutation status explain the clustering?

To assess whether common factors in melanoma cell lines and tumors explain the mixture model clustering, we performed logistic regression using BRAF and KRAS mutation status as predictors of cluster membership; BRAF and NRAS mutation status for the CCLE melanoma cell line cluster membership; and the BRAF V600E mutation status for the TCGA patient samples cluster membership. In each analysis, we found that the mutation status was not a significant predictor of the cluster membership (Table 2). For the CCLE cell lines, we also performed logistic regression with copy number variation, mutation, insertion, and deletion status as predictors of cluster membership. Interestingly, for the subset of melanoma cell lines in the CCLE data, the QQ-plots and FDR-adjusted *p*-values did not indicate any significant predictors of the cluster membership (Supplemental Figure 3C), although over the entire set of CCLE cell lines, the QQ-plots and FDR-adjusted *p*-values did suggest that genetic variations could predict or explain the two groups that were identified (Supplemental Figure 3D). These results were similarly observed in an analysis in which copy number variations (CNVs) were used as predictors of cluster membership (Supplemental Figure 7), suggesting that the gene expression differences identified across the entire CCLE cohort potentially arose from CNVs.

**Table 2:** Table of association test results between a priori called clusters and common factors in melanoma.

	**SU2C MAPK pathway cluster**				**TCGA MAPK pathway cluster**				**CCLE melanoma cell lines MAPK pathway cluster**			
**Mutation**	**Estimate**	**Std. error**	***z*-value**	**Pr(>|z|)**	**Estimate**	**Std. error**	***z*-value**	**Pr(>|z|)**	**Estimate**	**Std. error**	***z*-value**	**Pr(>|z|)**
BRAF+/–	0.2136	0.8554	0.25	0.8028					−0.2231	0.8878	−0.251	0.8016
KRAS+/–	0.3365	0.9562	0.352	0.72492								
BRAFv600e +/–					−0.1234	0.2786	−0.443	0.658				
NRAS+/–									−0.5522	0.9289	−0.594	0.552

*We test against available mutation status TCGA melanoma patients, SU2C melanoma cell lines, and CCLE melanoma cell lines*.

### Degree centrality analysis

**Figure 3:**
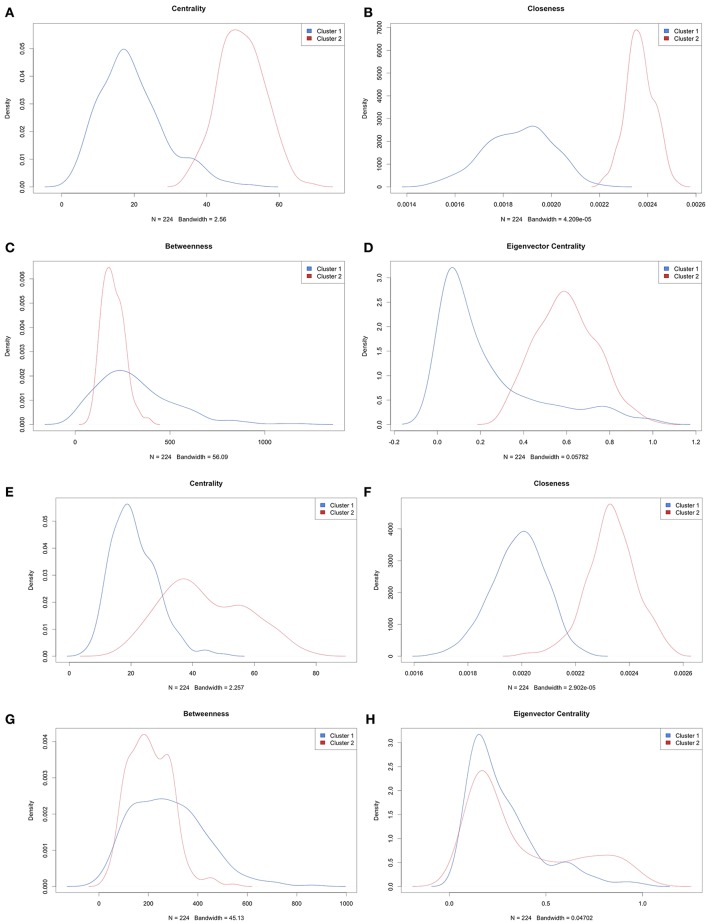
Density plots of degree centrality parameters. **(A–D)** are based on analysis of SU2C melanoma cell lines and **(E–H)** are based on analysis of CCLE melanoma cell lines. The plots are densities of centrality for **(A,E)**, closeness for **(B,F)**, betweenness for **(C,G)**, and Eigenvector centrality for **(D,H)**.

For each group or cluster identified from the multivariate mixture model (flexmix) analysis (for *k* = 2 groups), we assessed the statistical significance of the differences in pairwise gene expression correlations (where edges were kept if $\rho \left(r_{ij}^{2}\right)\leq 05$). We then removed all edges (i.e., gene pairs) whose correlation in expression levels was not statistically significant. We calculated the degree centrality statistics for those genes whose pairwise correlation strengths were statistically significant. The more connected or central genes were then extracted based on each degree centrality statistic and mapped to the Drug Gene Interaction Database (Table 3). This analysis suggested Bortezomib and Carfilzomib as potential drug targets in one of the clusters identified for each of the SU2C, CCLE melanoma, and all CCLE all cell lines. However, this same analysis applied to the second cluster for each of these data sets suggested that different drugs (Tofacitinib, AT9283, AZD1480, Pacritinib, Tofacitinib, and Citrate) would be better candidates, as least based on the connectedness or centrality of the genes given the identified transcriptional correlation strengths or networks. For the CCLE melanoma cell lines and the TCGA tumors, no drugs were identified based on this analysis for the second group. Finally, we compared the overall degree centrality statistics across all the data sets to assess their consistency (Figure 3). Density plots based on these statistics suggest that network rewiring was more pronounced in the SU2C melanoma cell lines and the CCLE melanoma cell lines, as there was a more obvious mixture of densities in these cohorts in the plots. The density plots for the TCGA samples and the complete set of cell lines for the CCLE data set were very similar and indicated that the differences in identified networks were again not as pronounced (Supplemental Figures 9,10). This is likely due to the heterogeneity between TCGA patient samples and cross-tissue pan-CCLE analysis (i.e., the cell lines are from different tissues, thus likely have very different underlying baseline networks).

**Table 3:** Network node centrality statistics identified central nodes for each parameter.

**Dataset**	**Centrality**	**Closeness**	**Betweenness**	**Eigenvector**	**Consensus**	**Predicted drug targets**
SU2C K1	201274_at (PSMA5)	201274_at (PSMA5)	201274_at (PSMA5)	201532_at (PSMA3)	PSMA5	BORTEZOMIB, CARFILZOMIB
SU2C K2	227677_at (JAK3)	227677_at (JAK3)	201648_at (JAK1)	227677_at (JAK3)	JAK3	TOFACITINIB, AT9283, AZD1480, PACRITINIB, TOFACITINIB CITRATE
CCLE Mel K1	201388_at (PSMD3)	201979_s_at (PPP5C)	201388_at (PSMD3)	201388_at (PSMD3)	PSMD3	BORTEZOMIB, CARFILZOMIB
CCLE Mel K2	226163_at (SYNGAP1, ZBTB9)	226163_at (SYNGAP1, ZBTB9)	207243_s_at (CALM2)	226163_at (SYNGAP1, ZBTB9)	SYNGAP1, ZBTB9	NA
CCLE All K1	201274_at (PSMA5)	201274_at (PSMA5)	201274_at (PSMA5)	201274_at (PSMA5)	PSMA5	BORTEZOMIB, CARFILZOMIB
CCLE All k2	208799_at (PSMB5)	208799_at (PSMB5)	208799_at (PSMB5)	201400_at (PSMB3)	PSMB5	BORTEZOMIB, CARFILZOMIB, OPROZOMIB
TCGA Mel K1	x819 (APBB1IP)	x14671 (RASGRF1)	x14671 (RASGRF1)	x819 (APBB1IP)	APBB1IP, RASGRF1	NA
TCGA Mel K2	x819 (APBB1IP)	x14632 (RAP1A)	x14632 (RAP1A)	x819 (APBB1IP)	APBB1IP, RAP1A	NA

*Consensus-based approach was used to genes to match to DGIdb to identify candidate therapeutic agents*.

### Are the networks equivalent for mutation status defined groups against normal controls?

We tested the possibility that network rewiring occurs as the consequence of mutation (i.e., the subgroups identified with supervised clustering were associated with the presence of a specific mutation in relevant cell lines). We first tested the equality of the correlation matrices derived from groups defined by mutation status for each data set defined by: BRAF+, BRAF−, KRAS+, and KRAS− against correlation matrices derived from skin fibroblast control gene expression data (as described in the Material and Methods section). Based on these matrix equivalency tests, we found evidence for statistically significant rewiring when comparing BRAF+, BRAF−, and KRAS− defined cell lines against skin fibroblast data (Table 4). However, when testing the KRAS+ against the control groups, the correlation matrix differences was not statistically significant from that derived from the skin fibroblast data (this may have been a result of the small sample size for the KRAS+ cell lines; *n* = 8).

**Table 4:** Table of correlation equality tests for the genes in the specified networks.

**Dataset**	**Samples**	**Network**	**St Chi-sq**	**Sim Mut St *p*-value**	**JR Chi-sq**	**Sim Mut JR *p*-value**	**Sig in 1 model?**
TGen cell line	BRAF+/–	BRAF GM	2.09E+02	**1.90E**−**02**	8.02E+02	7.54E−01	^*^
	BRAF+/control	BRAF GM	3.17E+02	**9.10E**−**03**	9.73E+02	2.68E−01	^*^
	BRAF−/Control	BRAF GM	3.34E+02	**3.10E**−**03**	6.71E+02	8.98E−01	^*^
	KRAS+/Control	BRAF GM	2.28E+02	1.33E−01	1.09E+03	6.02E−01	
	KRAS−/Control	BRAF GM	3.73E+02	**5.00E**−**04**	7.93E+02	3.41E−01	^*^
	KRAS+/–	KRAS GM	1.58E+02	4.37E−01	6.78E+02	**4.20E**−**02**	^*^
	BRAF+/control	KRAS GM	3.11E+02	**6.30E**−**03**	7.22E+02	8.20E−01	^*^
	BRAF−/Control	KRAS GM	2.86E+02	**1.48E**−**02**	7.07E+02	9.10E−01	^*^
	KRAS+/Control	KRAS GM	2.08E+02	2.41E−01	1.08E+03	7.45E−01	
	KRAS−/Control	KRAS GM	3.39E+02	**8.00E**−**04**	5.36E+02	9.53E−01	^*^
TCGA samples	BRAF+/–	BRAF GM	6.73E+02	1.81E−01	1.33E+03	**2.69E**−**02**	^*^
	BRAF V600E +/BRAFwt	BRAF GM	6.58E+02	2.26E−01	1.33E+03	**3.19E**−**02**	^*^
TCGA (WT somatic calls)	BRAF+/– (add somatic called)	BRAF GM	6.36E+02	3.11E−01	1.20E+03	3.59E−01	
	BRAF V600E +/BRAFwt	BRAF GM	6.25E+02	3.64E−01	1.24E+03	3.00E−01	
	BRAF V600E +/BRAFwt	26 DEGs GM	1.95E+04	1.89E−02	9.24E+04	9.99E−01	^*^

Tests are between mutant and wild type samples. Asterisks represent at least one significant test within comparison (Stieger or Jennrich).

*Bold values have an FDR adjusted p-value < 0.05*.

**Figure 4:**
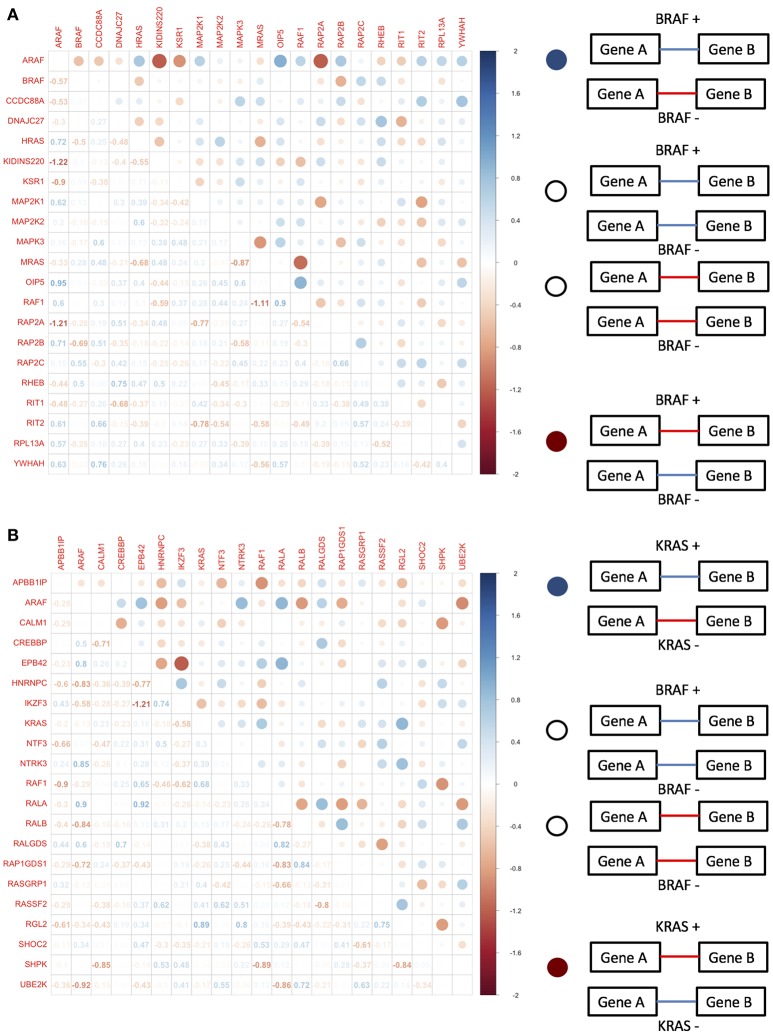
Differential pairwise correlation of **(A)** BRAF associated genes differentiated by BRAF mutation status and **(B)** KRAS associated genes differentiated by KRAS mutation status.

When we tested the equivalence of correlation matrices across BRAF and KRAS mutation status-defined groups within each data set, we found evidence for statistically significant differences based on tests that involved permuting mutation status (Table 4). We used differential correlations between mutant-defined and wild type-defined groups to assess the overall degree of rewiring. For the BRAF−associated genes, we found strong correlation differences between BRAF+ and BRAF− groups within a small set of gene pairs. However, for the KRAS−associated genes, roughly half of the genes pairs exhibited statistically significant correlation changes when assessing differences between KRAS+ and KRAS− groups (Figures 4A,B). Interestingly, when we considered the genes defined in the BRAF network for the TCGA melanoma samples, we also observed differences when comparing BRAF+/BRAF− derived correlation matrices as well as BRAF V600E+/BRAF− derived correlation matrices (Jennrich method significant, *p* < 0.027 and *p* < 0.032, respectively; Table 4). However, when we used VarScan to call the somatic mutation status and assign mutation status to the patient tumor samples, the signal from the matrix equivalence test was lost, suggesting that the network equivalence changes were only observable in the filtered samples.

### BRAF V600E+/– differentially expressed genes analysis with the melanoma TCGA tumor data

We identified genes that exhibited average gene expression level differences between BRAF V600E+ and BRAF− tumor samples in the entire TCGA data using DESeq (FDR adjusted *p* < 0.05) and edgeR (FDR < 0.01) between and identified 26 differentially expressed genes (DEGs) (Table 4). These genes were then used to extract a new gene network from geneMANIA. As anticipated, when we compared the correlation matrices based on the genes in this new network between BRAF+ and BRAF− groups, we observed statistically significant network changes. However, an analysis of the GO networks derived from these genes produced mostly non-significant differences between the BRAF V600E+ and BRAF− patient samples. These results indicate that the patient-specific network rewiring may have been isolated to specific nodes and were less pronounced than comparisons to the melanoma cell lines. To further investigate the overall differences in the correlations among gene expression levels between the TCGA BRAF V600E+/– samples, we calculated the spearman correlation between 348 DEGs (edgeR FDR < 0.01 and DESeq FDR-adjusted *p* < 0.05) and all other genes we had expression level data on. Using a strict Bonferroni-adjusted significance threshold, we observed 63,118 (0.88%) significant gene correlations in BRAF V600E+ patients, 81,765 (1.14%) significant gene correlations in BRAF− samples, and 21,844 (0.31%) significant gene pairs in common. Overall, we found that the majority of the significant gene pair correlations were significant in only one of the groups (Supplemental Figure 8), further indicating that the correlation strengths and overall transcriptional networks between TCGA BRAF V600E+ and BRAF− tumor samples are unique.

### Network centrality tests for the TGen melanoma cell lines

**Figure 5:**
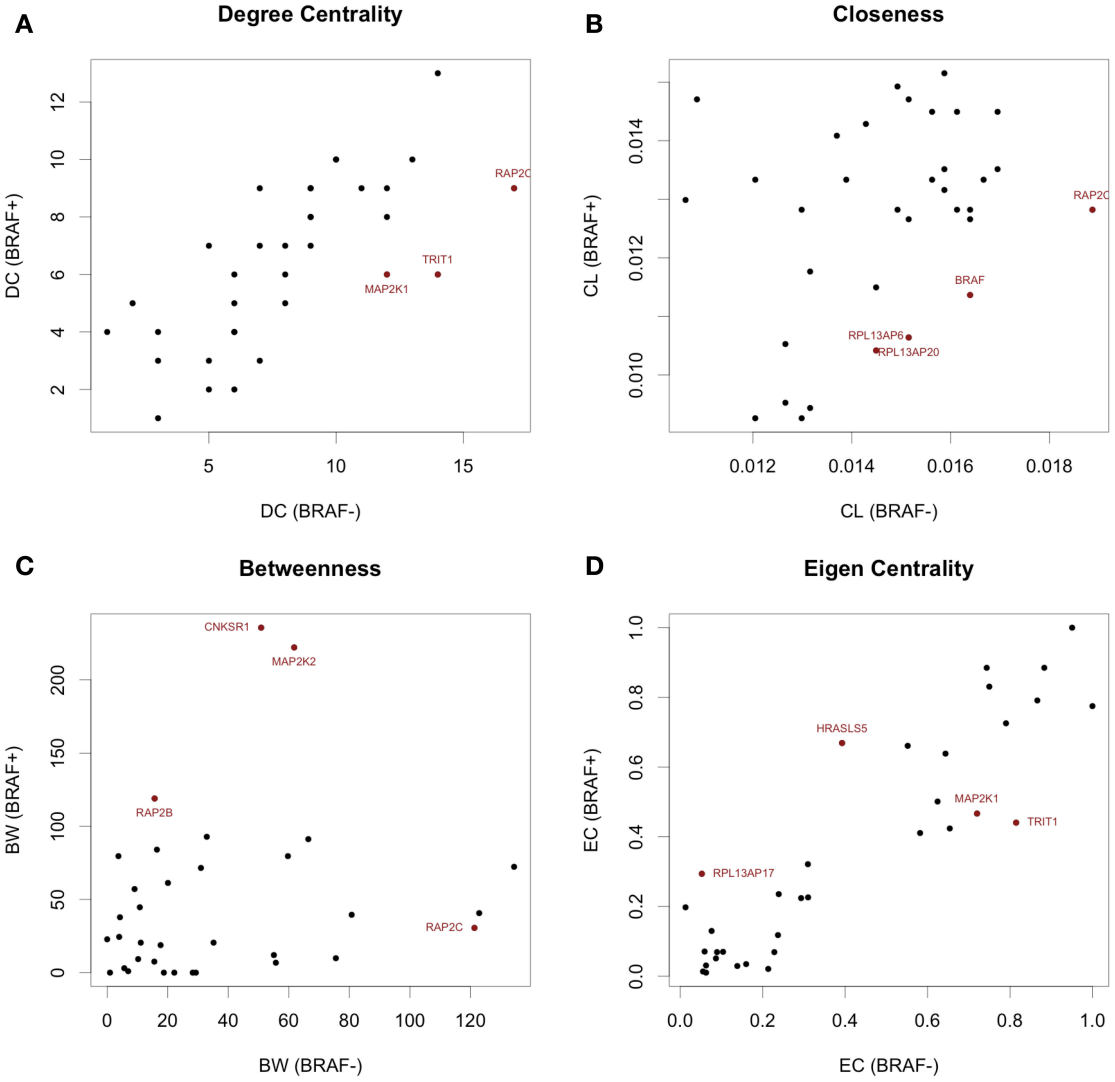
Network centrality statistics identifying differential central nodes between BRAF+ and BRAF− patients from TCGA data. **(A)** Degree Centrality, **(B)** Closeness Centrality, **(C)** Betweenness Centrality, and **(D)** Eigenvector Centrality can all be used to assess different properties of the network.

To investigate the importance of individual genes or pairs of genes in the transcriptional correlation networks we observed in our analyses of the TGen melanoma cell line data, we calculated the node centrality statistics for each network. These analyses suggested that the most important genes associated with BRAF+ cell lines for all genes in the BRAF network were ARAF, RAF1, and OIPS; whereas the most central or connected genes in the BRAF− cell lines were RAF1 and HRAS (Figures 5A,B, and Table 5). For the genes in the BRAF geneMANIA-derived network, the average correlation across all node centrality statistics was 0.1121 for BRAF+/− and 0.0001 for KRAS+/– indicating the existence of different central nodes (Tables 5, 6) for the networks. For the KRAS geneMANIA network, we observed negative correlations when comparing them across KRAS+/– cell lines for all node centrality statistics (Table 6), which indicates that extensive transcriptional rewiring is occurring in the networks. Additionally, based on the genes that appeared as most central to the identified networks, DGIdb analysis suggested different drug targets. For the BRAF+ cell lines, the most central or connected genes in the networks indicated that MEK inhibitors would be likely candidate drugs (Table 5). However, for the BRAF− cell lines, mTOR inhibitors were predicted as best targets.

**Table 5:** Assessing significant nodes by degree centrality parameters.

**Dataset**	**Samples**	**Network**	**DC**	**BW**	**CL**	**EC**	**Consensus**	**Predicted drug targets (top genes only)**
TGen Cell Line	BRAF+	BRAF GM	ARAF	ARAF	RAF1, ARAF, OIPS	ARAF	ARAF	Sorafenib, Trametinib, Regorafenib, BMS-908662, Adenosine Triphosphate
	BRAF−	BRAF GM	HRAS	RAF1, HRAS	RAF1, HRAS	HRAS	HRAS	AZD8055, Everolimus, DB08751
	KRAS+	KRAS GM	RASSF2, RGL2	RASSF2, RGL2	RASSF2, RGL2	RASSF2, RGL2	RASSF2, RGL2	
	KRAS−	KRAS GM	NTF3	NTF3	NTF3	NTF3	NTF3	
TCGA Samples	BRAF+	BRAF GM	CCDC88A	CNKSR1	CNKSR1	CCDC88A	CCDC88A, CNKSR1	
	BRAF V600E+	BRAF GM	CCDC88A	CNSKR1	RHEBL1	CCDC88A	CCDC88A	
	BRAF−	BRAF GM	RAP2C	RHEBL1	RAP2C	RAP2C	RAP2C	
	BRAF V600E+	26 DEGs GM	ATF1, CSTF3, GTG2E1, GFT2E2, GTF3A, PHTF2, TFEC, TFPI, TF, BCL2L15, FAM3C, PMCHL1	BCAP29	CSTF3	BCAP29	BCAP29	
	BRAF−	26 DEGs GM	TFPI	BCAP29	TFPI	BCAP29	TFPI, BCAP29	

*A consensus-based approach was used to genes to match to DGIdb to identify candidate therapeutic agents*.

**Table 6:** A comparison between degree centrality parameters through correlations.

**Dataset**	**Samples**	**Network**	**DC cor**	**BW cor**	**CL cor**	**EC cor**	**Avg cor**
TGen Cell Line	BRAF+/–	BRAF GM	0.05	0.15	0.10	0.14	0.11
TGen Cell Line	KRAS+/–	BRAF GM	0.10	0.03	0.00	−0.14	0.00
TGen Cell Line	BRAF+/–	KRAS GM	−0.06	−0.10	−0.36	−0.42	−**0.23**
TGen Cell Line	KRAS+/–	KRAS GM	−0.50	−0.19	−0.59	−0.32	−**0.40**
TCGA Samples	BRAF+/–	BRAF GM	0.77	0.26	0.28	0.85	0.54
TCGA Samples	BRAF V600E +/BRAFwt	BRAF GM	0.78	0.36	0.48	0.86	0.62
TCGA (WT somatic calls)	BRAF+/– (add somatic called)	BRAF GM	0.78	0.51	0.44	0.87	0.65
TCGA (WT somatic calls)	BRAF V600E +/BRAFwt	BRAF GM	0.79	0.68	0.65	0.85	0.74
TCGA (WT somatic calls)	BRAF V600E +/BRAFwt	26 DEGs GM	0.24	0.81	0.35	0.46	0.47

*DC, Degree Centrality; BW, Betweenness; CL, Closeness; EC, Eigenvector Centrality*.

*Bold values indicate negative average correlation*.

### Network centrality tests for the TCGA data

Centrality and connection analysis of the TCGA patient samples based on BRAF mutation status also identified distinct central genes in transcriptional networks. Although there was a strong positive correlation for each node centrality statistic (*r* > 0.48) across BRAF+ and BRAF V600E+ when compared against BRAF−, the networks derived from the gene expression-based correlation coefficients for BRAF+ patients had CCDC88A and CNKSR1 as most central or connected genes, whereas networks derived from BRAF V600E+ samples had only CCDC88A as the most important node (Figures 5C,D, and Table 5). CCDC88A is a key modulator of the AKT-mTOR signaling pathway (The UniProt Consortium, 2015) and CNKSR1 promotes invasion of cancer cells through NFκB dependent signaling (Fritz and Radziwill, 2010; Table 5). For the BRAF− derived networks, the central node was found to be RAP2C, which is a RAS-related protein in the MAPK/ERK signaling pathway. The consensus central nodes for melanoma cell lines with and without BRAF mutations were mapped to candidate drugs using DGIdb. Interestingly, RAF and MEK inhibitors (Sorafenib, Trametinib, Regorafenib, BMS-908662, and Adenosine Triphosphate) were predicted to be the best drugs given the observed BRAF+ transcriptional network, whereas the mTOR inhibitors (AZD8055, Everolimus, and DB08751) were predicted to be the best drugs in the observed BRAF− transcriptional network (Supplemental Figures 12–17, Table 5).

### Are strong edges present in both mutant and wild type groups?

To evaluate the strength of the correlation coefficients obtained, we assessed the overall distribution of coefficients (Supplemental Figure 18). We found that the distribution of pairwise correlation for BRAF−associated genes were approximately the same in both BRAF+ and BRAF− subgroups. However, when evaluating the KRAS−associated genes, there was an increased proportion of correlation coefficients near the tail for KRAS+ subgroup when compared against the KRAS− subgroup, which suggests a stronger network in the KRAS+ subgroup exists. This is further confirmed when assessing the upper 5% tail in correlation coefficients (for BRAF−associated genes, *r* = 0.54 in both BRAF+/BRAF− subgroups; for KRAS−associated genes, *r* = 0.46 in KRAS− subgroup and *r* = 0.76 for KRAS+ subgroup).

To assess whether the strongest co-expression patterns were observed in normal laboratory conditions, we extracted all correlation coefficients <−0.7 or >0.7 (Supplemental Table 2). We then used the resulting gene list as an input into geneMANIA in order in order to calculate the proportion of edges that were previously reported. We found that a majority of the strong co-expression edges were not previously identified (Supplemental Figure 19). Specifically, for the BRAF− subgroup, RAP2B and RAP2C are co-localized and share protein domains; for the BRAF+ subgroup, BRAF and RHEB are in the same pathway and have physical interactions; and for the KRAS+ subgroup, RALA and UBE2K are co-expressed, KRAS and RASSF2 have physical interactions, RGL2 and KRAS are in the same pathway and have physical interactions, and RASSF2 and APBB1IP have shared protein domains. Thus, only six of the 24 identified strong edges were previously reported. This low number of replicated edges could in fact be due, in part, to sampling and statistical artifacts having to do with, e.g., the low expected correlation under equilibrium states due to range restriction effects (Giuliani et al., 2004; Gorban et al., 2010). In fact, the finding that the strongly correlated genes are not observed under equilibrium states further indicates the need for using unsupervised approaches since such approaches would not require imposing assumptions about the factors or network topology that drive disease pathogenesis.

### *De novo* network reconstruction analysis

**Figure 6:**
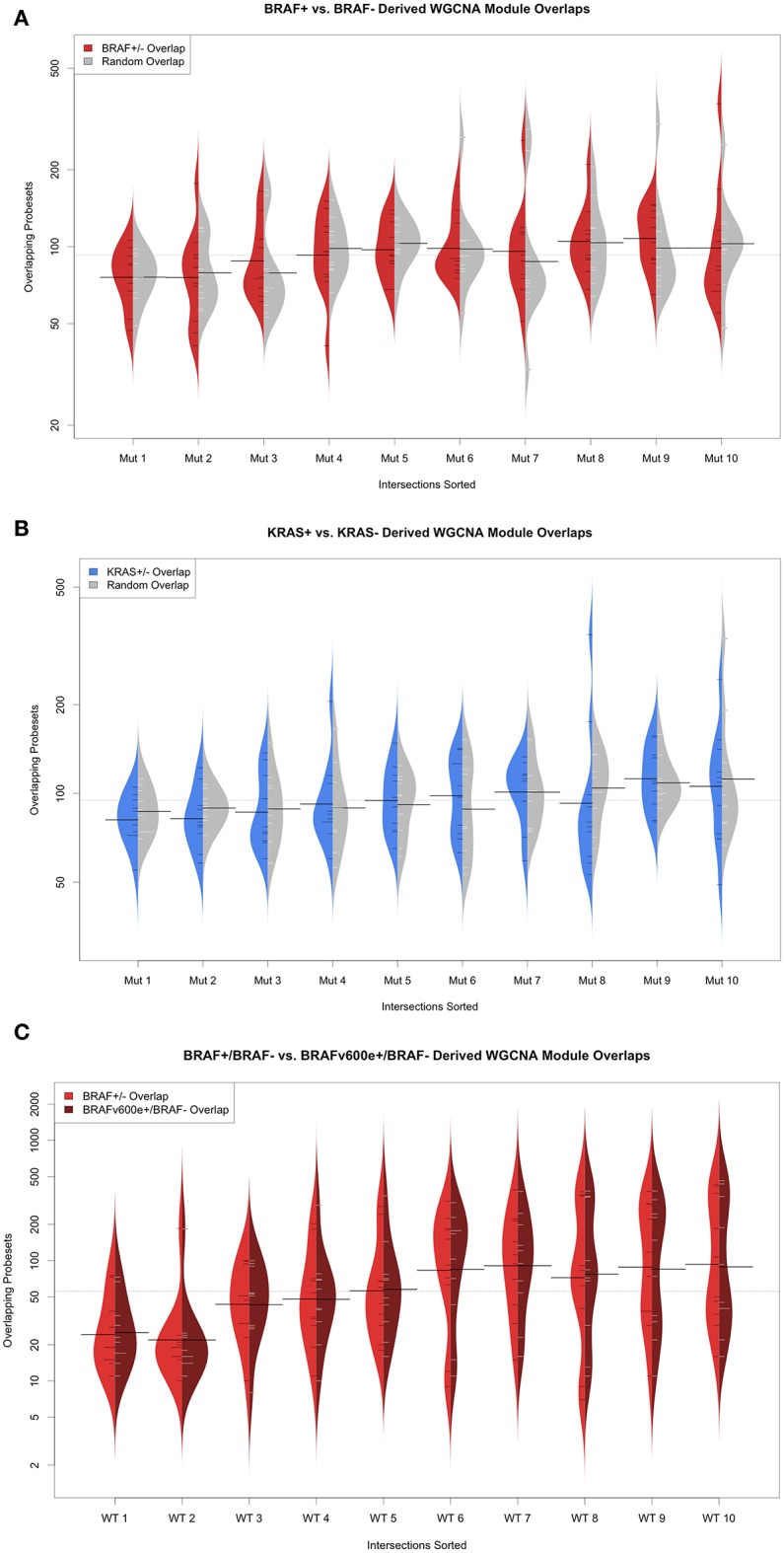
WGCNA derived modules used for *a priori* assessment of network rewiring. **(A)** Beanplots of overlapping modules between melanoma cell line BRAF+/− (red) and random permutation-based (gray) overlap **(B)** Beanplots of overlapping modules between melanoma cell line KRAS+/− (blue) and random permutation-based (gray) and **(C)** Beanplots of overlapping modules between TCGA melanoma patient BRAF+/− (red) and BRAFv600e+/− (dark red) overlap.

We also considered reconstructing transcriptional networks *de novo* using WCGNA (Gautier et al., 2004; Barrett et al., 2013) across mutation-status (supervised) groups on the TGen melanoma cell lines and TCGA melanoma patient samples. For the TGen melanoma cell lines, WGCNA modules were created using BRAF and KRAS mutation groupings (Figure 6). For each of the modules identified from the WCGNA assessment of the wild-type cell lines, we identified the modules identified in the mutation-bearing cell lines with the highest intersection of genes. We then tested these intersecting genes for network rewiring using network equivalence and permutation tests (although we were unable to test for KRAS because of the limited number of KRAS+ melanoma cell lines). Our results confirmed evidence for network rewiring based on these *de novo* reconstructed networks (Table 7). When creating 10 modules for the melanoma cell lines, six out of the 10 networks had strong *p*-values; and when creating 20 modules for the melanoma cell lines, seven out of the 20 networks had strong *p*-values. Although the network rewiring in the TCGA patients was not as extensive, there were still some modules that had statistically significant *p*-values (Supplemental Figure 17 and Table 7). We also found that there was network rewiring in a small number of *a priori* created modules in both BRAF+ vs. BRAF− and BRAFV600E+ vs. BRAF−- melanoma cohorts.

**Table 7:** Correlation equality tests between WGCNA defined modules in BRAF+/− SU2C cell lines, BRAF+/− TCGA melanoma patients, and BRAFv600e+/− TCGA melanoma patients.

**SU2C cell lines: BRAF**+ **vs. BRAF**−				**TCGA melanoma patients: BRAF**+ **vs. BRAF**−				**TCGA melanoma patients:** **BRAFv600e**+ **vs. BRAF**−			
**WGCNA module**	**Size**	**St Chi-sq**	***P*-value**	**WGCNA module**	**Size**	**St Chi-sq**	***P*-value**	**WGCNA module**	**Size**	**St Chi-sq**	***P*-value**
WGCNA 1 (*k* = 10)	177	21,137	0.003	WGCNA 1 (*k* = 10)	388	104,851	0.055	WGCNA 1 (*k* = 10)	375	90,565	0.125
WGCNA 2 (*k* = 10)	210	22,813	0.152	WGCNA 2 (*k =* 10)	374	80,097	0.251	WGCNA 2 (*k =* 10)	379	81,201	0.262
WGCNA 3 (*k =* 10)	363	NA		WGCNA 3 (*k =* 10)	311	46,891	0.598	WGCNA 3 (*k =* 10)	304	46,141	0.512
WGCNA 4 (*k =* 10)	173	21,100	0.001	WGCNA 4 (*k =* 10)	203	26,916	0.132	WGCNA 4 (*k =* 10)	289	59,361	0.125
WGCNA 5 (*k =* 10)	146	9,770	0.135	WGCNA 5 (*k =* 10)	375	103,329	0.130	WGCNA 5 (*k =* 10)	380	106,081	0.128
WGCNA 6 (*k =* 10)	151	12,584	0.042	WGCNA 6 (*k =* 10)	100	5,848	0.082	WGCNA 6 (*k =* 10)	100	**7,580**	**0.002**
WGCNA 7 (*k =* 10)	139	10,240	0.044	WGCNA 7 (*k =* 10)	432	110,980	0.228	WGCNA 7 (*k =* 10)	462	133,054	0.167
WGCNA 8 (*k =* 10)	106	6,058	0.002	WGCNA 8 (*k =* 10)	75	**4,025**	**0.042**	WGCNA 8 (*k =* 10)	73	**4,256**	**0.024**
WGCNA 9 (*k =* 10)	261	27,640	0.343	WGCNA 9 (*k =* 10)	282	**54,269**	**0.042**	WGCNA 9 (*k =* 10)	346	79,352	0.063
WGCNA 10 (*k =* 10)	165	16,857	0.069	WGCNA 10 (*k =* 10)	185	18,780	0.381	WGCNA 10 (*k =* 10)	184	18,864	0.337
WGCNA 1 (*k =* 10)	115	6,637	0.183	WGCNA 1 (*k =* 20)	127	9,636	0.123	WGCNA 1 (*k =* 20)	128	10,610	0.067
WGCNA 2 (*k =* 20)	86	2,174	0.987	WGCNA 2 (*k =* 20)	161	13,476	0.500	WGCNA 2 (*k =* 20)	159	12,823	0.513
WGCNA 3 (*k =* 20)	41	868	0.121	WGCNA 3 (*k =* 20)	139	8,824	0.712	WGCNA 3 (*k =* 20)	136	8,577	0.712
WGCNA 4 (*k =* 20)	50	1,693	0.007	WGCNA 4 (*k =* 20)	160	12,930	0.457	WGCNA 4 (*k =* 20)	140	10,143	0.419
WGCNA 5 (*k =* 20)	38	731	0.089	WGCNA 5 (*k =* 20)	138	10,935	0.226	WGCNA 5 (*k =* 20)	118	8,565	0.206
WGCNA 6 (*k =* 20)	58	1,269	0.563	WGCNA 6 (*k =* 20)	405	112,288	0.142	WGCNA 6 (*k =* 20)	370	89,286	0.184
WGCNA 7 (*k =* 20)	172	11,040	0.481	WGCNA 7 (*k =* 20)	201	35,418	0.082	WGCNA 7 (*k =* 20)	181	28,132	0.095
WGCNA 8 (*k =* 20)	38	834	0.053	WGCNA 8 (*k =* 20)	105	7,231	0.125	WGCNA 8 (*k =* 20)	159	14,801	0.246
WGCNA 9 (*k =* 20)	58	2,249	0.017	WGCNA 9 (*k =* 20)	171	17,254	0.256	WGCNA 9 (*k =* 20)	173	17,306	0.274
WGCNA 10 (*k =* 20)	48	881	0.400	WGCNA 10 (*k =* 20)	187	20,292	0.217	WGCNA 10 (*k =* 20)	189	21,525	0.185
WGCNA 11 (*k =* 20)	88	4,670	0.150	WGCNA 11 (*k =* 20)	69	3,021	0.111	WGCNA 11 (*k =* 20)	64	2,668	0.098
WGCNA 12 (*k =* 20)	124	NA		WGCNA 12 (*k =* 20)	246	34,917	0.266	WGCNA 12 (*k =* 20)	222	28,240	0.272
WGCNA 13 (*k =* 20)	35	971	0.003	WGCNA 13 (*k =* 20)	55	1,602	0.372	WGCNA 13 (*k =* 20)	54	1,688	0.201
WGCNA 14 (*k =* 20)	88	3,814	0.215	WGCNA 14 (*k =* 20)	218	26,418	0.330	WGCNA 14 (*k =* 20)	230	27,212	0.469
WGCNA 15 (*k =* 20)	50	2,179	0.016	WGCNA 15 (*k =* 20)	21	**294**	**0.024**	WGCNA 15 (*k =* 20)	15	**175**	**0.018**
WGCNA 16 (*k =* 20)	46	1,193	0.049	WGCNA 16 (*k =* 20)	134	12,283	0.161	WGCNA 16 (*k =* 20)	84	4,155	0.289
WGCNA 17 (*k =* 20)	45	1,638	0.023	WGCNA 17 (*k =* 20)	128	10,301	0.227	WGCNA 17 (*k =* 20)	153	15,412	0.192
WGCNA 18 (*k =* 20)	72	3,108	0.077	WGCNA 18 (*k =* 20)	170	15,248	0.420	WGCNA 18 (*k =* 20)	169	15,297	0.416
WGCNA 19 (*k =* 20)	41	1,211	0.016	WGCNA 19 (*k =* 20)	117	8,769	0.236	WGCNA 19 (*k =* 20)	115	9,200	0.134
WGCNA 20 (*k =* 20)	42	1,030	0.056	WGCNA 20 (*k =* 20)	12	89	0.092	WGCNA 20 (*k =* 20)	13	77	0.509

*Bold values have an FDR adjusted p-value < 0.05*.

## Discussion

Although there has been a great deal of attention given to the development and use of pathway and genetic network analysis tools in understanding disease pathogenesis and drug targeting, these tools often rely upon the use of pathway and network information derived from analyses of genes in normal and non-diseased cells, cell lines, and tissues (St Onge et al., 2007; Califano, 2011; Ideker and Krogan, 2012; Hofree et al., 2013). This compromises relevant analyses since pathways and genetic networks are not static and are often perturbed by various diseases of interest. This is especially true in the study of cancer, where there are many stresses, including gene perturbations, drugs and environmental influences, that all impact the way genes interact and exploit, e.g., signaling or regulatory relationships. This is not to say that researchers cannot use information about the relationships among genes derived from studies of normal cells and tissues as an initial starting point for detecting differences in gene relationships in a disease state, but it does suggest that a great deal of caution should be made in making claims about the relationships between genes in a disease state, i.e., their wiring or topology as reflected in the networks they form.

We assessed evidence for network rewiring *a posterori* among a set of melanoma cell lines and tumor profiles by leveraging multivariate mixture models in the analysis of the relationships among gene expression values. The mixture models allowed us to identify subsets of cell lines and tumors that exhibit the most evidence for rewiring (i.e., differences in the relationships among genes based on their expression patterns) without having to specify *a priori* which cell lines or tumors we wanted to contrast for network differences. We focused on the MAPK pathway in particular and found that, based on very robust simulation-based tests, heterogeneity in networks reflective of rewiring did indeed exist in our cell lines and tumor profiles. Furthermore, we explored various factors that could explain the differences between the identified cell lines, tumor profiles identified from the mixture models, but found that, in the melanoma cell lines, common melanoma gene alterations could not explain the group differences we identified, suggesting that some other factor is responsible for the rewiring in these groups of cell lines and tumors. We also applied degree centrality and other network node importance statistics to the networks in the subgroups of cell lines and tumors identified. These analyses suggested that different genes played more central roles in the different subgroup networks and hence are likely better drug targets. These results suggest that the use of pathway and genetic network information on a *biologically relevant* subset of cell lines or tumors may reveal greater insights into drug candidates.

As a complement to using mixture models to identify novel subgroups of melanoma cell lines and tumors exhibiting evidence for genetic network rewiring, we also compared the genetic networks based on correlations between expression levels of genes in melanoma cell lines and patient samples across groups defined by the presence of specific mutations in supervised analyses. We found that there was evidence of network rewiring when stratifying the cell lines based on mutation status. Although network rewiring was also observed in the patient samples, it was less pronounced. The cancer cell lines may have gone through numerous passages, contributing to changes in genetic network and gene expression patterns. Another potential explanation is that cell lines represent a fairly homogeneous population with chaotic genomes, while patient tumor samples are heterogeneous with respect to the mutations they carry and are often comprised of tumor, stroma, infiltrating immune cells, and other non-tumor cells. It is likely that this heterogeneity makes it much more difficult to clearly identify changes in the correlations in the expression levels of genes. Tumor heterogeneity also makes identifying consensus between-patient drug targets more difficult since patient-specific rewirin” could affect the downstream drug target identification.

Our analyses also involved the use of various network centrality statistics to identify candidate genes (i.e., nodes in the networks) that might be ideal drug targets among both BRAF mutant cell lines and BRAF wild-type cell lines, especially for groups defined (in a supervised manner) by mutation status. As expected, we found that MEK inhibitors were the most likely drugs to affect BRAF mutant cell lines, whereas the mTOR inhibitors were the most likely drugs to affect BRAF wild-type cell lines. Ultimately, we feel that our analyses strongly suggest that an understanding of how the relationships among genes or other factors influencing, or associated with, disease pathogenesis can facilitate treatment decisions. However, the identification of these relationships requires a clear sensitivity to the fact that those relationships change in the disease state in ways that cannot be easily anticipated from, e.g., mutation status or average gene expression levels alone.

## Materials and methods

### Transcriptional profiles on cell lines

We leveraged a number of melanoma cell lines. Including the UACC cell lines maintained at the Translational Genomics Research Institute (TGen) in Phoenix, AZ. All the TGen UACC cell lines were of low passage number (Figure 1B) and were maintained according to the manufacturer's or collaborator's instructions. All cell lines were stored and evaluated in media with 10% FBS and 1% AA added. The low passage cell lines were maintained in multiple aliquots as liquid nitrogen stocks to reduce risk of phenotypic drift. All cells were cultured for less than 3 months before reinitiating culture from the frozen stock and routinely inspected for identity by morphology and growth curve analysis and validated to be mycoplasma free. All cell lines were free of contaminants.

The cell line samples were used for nucleic acid extraction, verification, amplification, and hybridisation to Affymetrix HG-U133 plus 2.0 arrays (54,675 probesets, Affymetrix, Santa Clara, CA) using standard protocols for Affymetrix GeneChip microarrays. Affymetrix HG-U133 Plus 2.0 microarrays were normalized (background adjustment, interquartile normalization, and median polish) using robust multichip averaging (Irizarry et al., 2003) in R. To assess network rewiring that occurs within melanoma cell lines against a control group, we normalized expression profiles across melanoma cell line and skin fibroblast samples (Supplemental Figure 1, Supplemental Table 1). All subsequent analyses were based on normalization only within melanoma cell lines (Supplemental Figure 2).

We also obtained the SK-MEL-2 melanoma cell line from the Cancer Cell Line Encyclopedia (Barretina et al., 2012) (CCLE), and the MeWo melanoma cell line from the Developmental Therapeutics Program's NCI-60. In addition, we leveraged microarray gene expression data on skin fibroblast samples (*n* = 29) from Gene Expression Omnibus (Edgar et al., 2002; Barrett et al., 2013) (GEO) (Supplemental Table 1). The skin fibroblast samples varied in age and ethnicity (Caucasian and Puerto Rican). Finally, we obtained gene expression data from the TCGA melanoma samples from the Broad GDAC Firehose (*n* = 474). Raw CEL files were downloaded from the CCLE web portal. All samples from the CCLE, NCI-60, and GEO were performed on Affymetrix HG-U133 plus 2.0 arrays. All microarray samples were re-normalized together to ensure minimal batch effects. Normalization of all microarray data (background adjustment, interquartile normalization, and median polish) was completed using robust multichip averaging (Gautier et al., 2004) in R. We obtained RAF and RAS mutation status on the CCLE cell lines from the Oncomap mutation provided in the CCLE web portal and additional binary calls for copy number and mutation data from the CCLE web portal.

### MAPK pathway information

We took advantage of a wide variety of data analysis techniques, as described in detail below but focused much of our analyses on sets of genes in the MAPK pathway, which were extracted from the information in the WikiPathways (Gorban et al., 2010; Censi et al., 2011) resource. We ultimately matched MAPK pathway genes to probe sets in the Affymetrix arrays used on for transcriptional profiling on the cell lines. We also matched MAPK genes to those evaluated in TCGA RNAseq dataset. For genes with multiple probe set matches, the probe set with the maximum average intensity was used. We fit multivariate mixture models using flexmix (Leisch, 2004; Gruen and Leisch, 2007, 2008), assuming that there were two (BRAF+ vs BRAF−) or three (possible sub-grouping for BRAF−) overarching different groups with gene expression values as the dependent variables.

### Unsupervised network rewiring analysis

In order to conduct analyses exploring evidence for network heterogeneity without specifying a grouping factor *a priori* (i.e., in an unsupervised manner) we used multivariate mixture models as implemented in the R module flexmix (Fritz and Radziwill, 2010; Gorban et al., 2010; The UniProt Consortium, 2015). For the multivariate mixture models, we hypothesized that groups identified in the data had different covariance (correlation) structures reflecting the strength of the associations of the expression values of genes within networks. We tested for the existence of two groups in the data, but our approach could have easily tested for three or more groups; however, sample size limitations would have resulted in poor power for such analyses. Based on the patterns of the correlations between expression levels of the genes within each group, we sought to identify blocks of highly correlated genes by using simple hierarchical clustering of the pairwise correlations. Dendrograms or trees reflecting this clustering could then be cut at varying heights to assess the extent of heterogeneity within the flexmix analysis-derived groups.

### Assessing statistical significance

In order to determine whether there was a significant statistical evidence for subgroups within a data set regulated exhibiting different transcriptional networks, we leveraged simulation-based tests. For a given analysis, we simulated 1,000 samples with an *n*-dimensional mean vector and an *n* x *n* covariance matrix consistent with the observed mean vectors and covariance matrices for the full data set (i.e., under the assumption of homogeneity or non-subgroups). We then used flexmix to identify two subgroups with these simulated data sets. From these flexmix analyses, we calculated t-statistics comparing the estimated mean vectors, chi-square statistics comparing correlation matrices, as well as log-likelihood ratios, delta AICs, and delta BICs comparing the two group vs. single group fits. We calculated *p*-values for the statistics obtained from the non-simulated data as the number of simulated sample statistics greater than the actual test statistics divided by 1,001.

### *A posteriori* supervised analyses

To determine whether or not the two groups identified in an analysis could be explained by the existence of gene mutations present or not in the cell lines, we performed logistic regression analysis with the flexmix group identifier for a cell line as the dependent variable and BRAF, NRAS, and KRAS mutation information on those cell lines as explanatory variables. For the CCLE cell lines, additional association tests were pursued using gene mutation, gene amplification, insertion and deletion information as independent variables. To account for the multiple hypothesis tests, we used Q:Q-plots and Benjamini-Hochberg (Benjamini and Hochberg, 1995) FDR-adjusted *p*-values.

### Exploring network architecture

To determine whether particular genes exhibited stronger correlations with other genes and were acting as potential “hubs” within the transcriptional networks, we calculated network centrality metrics for each gene. To do this, individual “edges” between each pair of nodes (*i, j*; where *i* and *j* denote genes or nodes in the transcriptional network) were set equal to:

$$E_{ij}=\{\begin{array}{c} 0,\;if\rho \left(r_{ij}^{2}\right)>0.05\\ r_{ij}^{2},\;otherwise \end{array}$$

where $r_{ij}^{2}$ reflects the correlation between genes *i* and *j* and $\rho \left(r_{ij}^{2}\right)$ is the *p*-value associated with that correlation, to create graphs of the network. These calculations were performed for each group identified from the mixture analysis using flexmix. Based on the resulting graphs, we then calculated the metrics degree centrality, closeness centrality, betweenness centrality, and eigenvector centrality for each gene within each group's network. With these metrics for each gene, we examined the degree of similarity of genes across groups (i.e., we compared the metrics for each gene across the mixture model-derived groups). For those genes that emerged as the most central for each network, we identified drugs that target those genes using the Drug Gene Interaction Database (DGIdb).

### Analyses of the CCLE melanoma cell line expression data

To assess evidence for network rewiring associated with groups of tumors defined by mutational status (i.e., a supervised analysis), we tested the equality of correlation matrices computed for each group using correlation matrix equivalence tests. Specifically, we extracted gene networks centered on BRAF and KRAS genes using geneMANIA. For this analyses, we identified the 20 most inter-connected genes associated with the BRAF and KRAS pathways. Although NRAS is a more common mutation in melanomas, we pursued more focused analysis on the KRAS mutation because the cell lines and patient splits had a greater proportion of KRAS mutant samples (i.e., there were enough KRAS samples to pursue an analysis, but only a limited number of NRAS mutant samples). We then calculated the pairwise spearman correlations for the gene expression values for those genes within mutant melanoma cell lines and wild type melanoma cell lines. We compared the resulting correlation matrices through permutation-based tests (see above). We calculated χ^2^-statistics using the Steiger and Jennrich correlation equivalence test methods with the psych (Revelle, 2015) package in R. As noted in the package manual, the Steiger method uses the sum of the squared correlations, whereas the Jennrich method compares the differences between two matrices to the averages of the two matrices. Thus, the Steiger method emphasizes non-zero values to a greater degree, whereas the Jennrich method places a greater emphasis the change in correlations. We generated null distributions of χ^2^-statistics through permutations of the mutation status. For each test, we randomly permuted mutation status 10,000 times, repeated the analyses, and derived the distribution of test statistics from these permutations to get a null distribution as well as *p*-values for χ^2^-statistics.

### Analyses of the TCGA melanoma tumor gene expression data

We performed similar correlation and permutation-based tests within the TCGA melanoma gene expression data. For each melanoma patient's tumor gene expression data in the TCGA, we obtained expression profiles from normal and tumor from Cancer Genomics Hub. We used BAM slicer to identify BRAF and KRAS gene regions and we assigned somatic mutation status using the default settings in VarScan. We stratified patient samples into three groups: BRAF mutant (BRAF+), BRAF V600E mutant (BRAF V600E+), and BRAF wildtype (BRAF−). We then performed correlation equivalence tests to compare BRAF+ against BRAF− derived spearman correlation matrices and BRAF V600E+ against BRAF− derived spearman correlation matrices. As before, we generated null distributions of relevant χ^2^-statistics by permuting the mutation status. To determine whether evidence for rewiring was limited to the extracted BRAF network, we explored evidence for broader network differences by identifying genes that exhibited differential expression between mutant and wild type groups using the DESeq and edgeR packages in R. We used these genes as inputs to identify candidate networks by assessing them with geneMANIA and GO. Resulting networks were then assessed for differences across the mutation-defined groups, using correlation equivalence tests and degree centrality statistics on these additional DEG-derived networks.

### Network reconstruction analyses

As a complement to analyses comparing correlation matrices, we used the Weighted Gene Correlation Network Analysis (Langfelder and Horvath, 2008, 2012) (WGCNA) package in R to identify significant transcriptional modules within mutation bearing and non-mutation-bearing samples. We created modules (setting WCGNA parameter *k* to *k* = 10 and *k* = 20) within BRAF+/- and KRAS+/- groups for the SU2C melanoma cell lines; and BRAF+/- and BRAF V600E+/BRAF− for the TCGA patient tumor samples. Corresponding modules were defined as the modules with the maximum overlapping gene matches between BRAF− or KRAS− modules and BRAF+, BRAF V600E+, or KRAS+ modules. For the intersections of genes identified, we assessed network rewiring using the previously mentioned node centrality statistics and correlation equivalence tests.

## Author contributions

K-FD performed all analysis, generated figures and tables, and wrote the manuscript. DF, HY, WH, CS, JK, and AS performed the cell line drug response screens and gene expression assays. WH generated Figure 1B; PL, KV, JT, and NS wrote proposals for the study and coordinated all aspects of the study. NS provided key scientific discussion and guidance around the statistical methods used. edited and revised the manuscript and overall support around this research.

### Conflict of interest statement

The authors declare that the research was conducted in the absence of any commercial or financial relationships that could be construed as a potential conflict of interest.

## Supplementary material

Supplementary Figure 1 Boxplot of normalized gene expression profiles across all melanoma cell lines and skin fibroblast samples.

Supplementary Figure 2 Boxplot of normalized gene expression profiles within only melanoma cell lines.

Supplementary Figure 3 Density plots for the posterior probability of the flexmix groups in **(A)** SU2C melanoma cell lines and **(B)** CCLE melanoma cell lines. These plots reveal that the flexmix called clusters were most distinct in the SU2C melanoma cell lines. **(C,D)** are QQ-plots for gene significance association tests against melanoma cell line groups discovered in the CCLE data.

Supplementary Figure 4 Circular network graph of all common nodes. Top network represents the network derived from correlation associations found in cluster one, whereas the bottom network represents the edges present in cluster two. Density of the edges highlights the differences in networks.

Supplementary Figure 5 Pairwise correlation between MAPK Pathway genes from the identified clusters in TCGA melanoma patients using flexmix were used to generate heatmaps. Upper right hand half of matrix were correlation coefficients from cluster 1, whereas lower left hand half of matrix were correlation coefficients from cluster 2. Hierarchical clustering was subsequently performed using either distances from cluster 1 coefficients **(A)** or cluster 2 coefficients **(B)**.

Supplementary Figure 6 Pairwise correlation between MAPK Pathway genes from the identified clusters in all CCLE cell lines using flexmix were used to generate heatmaps. Upper right hand half of matrix were correlation coefficients from cluster 1, whereas lower left hand half of matrix were correlation coefficients from cluster 2. Hierarchical clustering was subsequently performed using either distances from cluster 1 coefficients **(A)** or cluster 2 coefficients **(B)**.

Supplementary Figure 7 QQ-plots for *p*-values based on association tests for gene copy number variation observed against the groups discovered in the CCLE dataset, using either the melanoma subset **(A,B)** or against all cell lines **(C,D)**.

Supplementary Figure 8 Observed *p*-values based on correlation strength tests. Correlations were compared between those derived from TCGA melanoma patients with BRAFv600e mutation against those derived from BRAF wt. Blue points represent edges significant in only BRAF−, red points represent points significant in only BRAFv600e+, black points represent significance in both.

Supplementary Figure 9 Density plot of node centrality statistics for flexmix clusters called on TCGA melanoma patients. We use four different degree centrality parameters **(A)** Centrality, **(B)** Closeness, **(C)** Betweenness, and **(D)** Eigenvector Centrality.

Supplementary Figure 10 Density plot of node centrality statistics for flexmix clusters called across all CCLE cell lines. We use four different degree centrality parameters **(A)** Centrality, **(B)** Closeness, **(C)** Betweenness, and **(D)** Eigenvector Centrality.

Supplementary Figure 11 Comparing node centrality statistics for BRAF+ and BRAF− from the SU2C cell lines. We use four different degree centrality parameters **(A)** Centrality, **(B)** Closeness, **(C)** Betweenness, and **(D)** Eigenvector Centrality.

Supplementary Figure 12 Comparing node centrality statistics for KRAS+ and KRAS− from the SU2C cell lines. We use four different degree centrality parameters **(A)** Centrality, **(B)** Closeness, **(C)** Betweenness, and **(D)** Eigenvector Centrality.

Supplementary Figure 13 Comparing node centrality statistics for BRAFv600e+ and BRAF− from the TCGA melanoma patients. We use four different degree centrality parameters **(A)** Centrality, **(B)** Closeness, **(C)** Betweenness, and **(D)** Eigenvector Centrality.

Supplementary Figure 14 Network derived from edges present in TCGA BRAF+ patients.

Supplementary Figure 15 Network derived from edges present in TCGA BRAFv600e+ patients.

Supplementary Figure 16 Network derived from edges present in TCGA BRAF− patients.

Supplementary Figure 17 WGCNA derived modules (with k = 20) used for *a priori* assessment of network rewiring. **(A)** Beanplots of overlapping modules between melanoma cell line BRAF± (red) and random permutation-based (gray) overlap **(B)** Beanplots of overlapping modules between melanoma cell line KRAS± (blue) and random permutation-based (gray) and **(C)** Beanplots of overlapping modules between TCGA melanoma patient BRAF± (red) and BRAFv600e± (dark red) overlap.

Supplementary Figure 18 Density plots of the observed pairwise correlation coefficients when comparing **(A)** the BRAF+/− subgroup using the BRAF−associated gene network and **(B)** the KRAS+/− subgroup using the KRAS−associated gene network.

Supplementary Figure 19 GeneMANIA circular view of all genes in Supplemental Table 2, i.e., genes with strong interactions identified via co-expression analysis.

Supplementary Table 1 List of skin fibroblast samples obtained from GEO. Sample repository, sample name, and associated meta data is provided.

Supplementary Table 2 Identifying edges within BRAS-associated gene networks and KRAS−associated gene networks using co-expression observed within each subgroup. Edges reported had a correlation coefficient <−0.7 or >0.7.
