# Supplementary material for: Network Rewiring in Cancer: Applications to Melanoma Cell Lines and the Cancer Genome Atlas Patients
Source: Front Genet. 2018 Jul 10;9:228. doi: 10.3389/fgene.2018.00228 (PMC6048451; doi:10.3389/fgene.2018.00228)
Supplement: Supplementary Figure 1 — Boxplot of normalized gene expression profiles across all melanoma cell lines and skin fibroblast samples. [file Presentation_1.PPTX]

## Slide 1
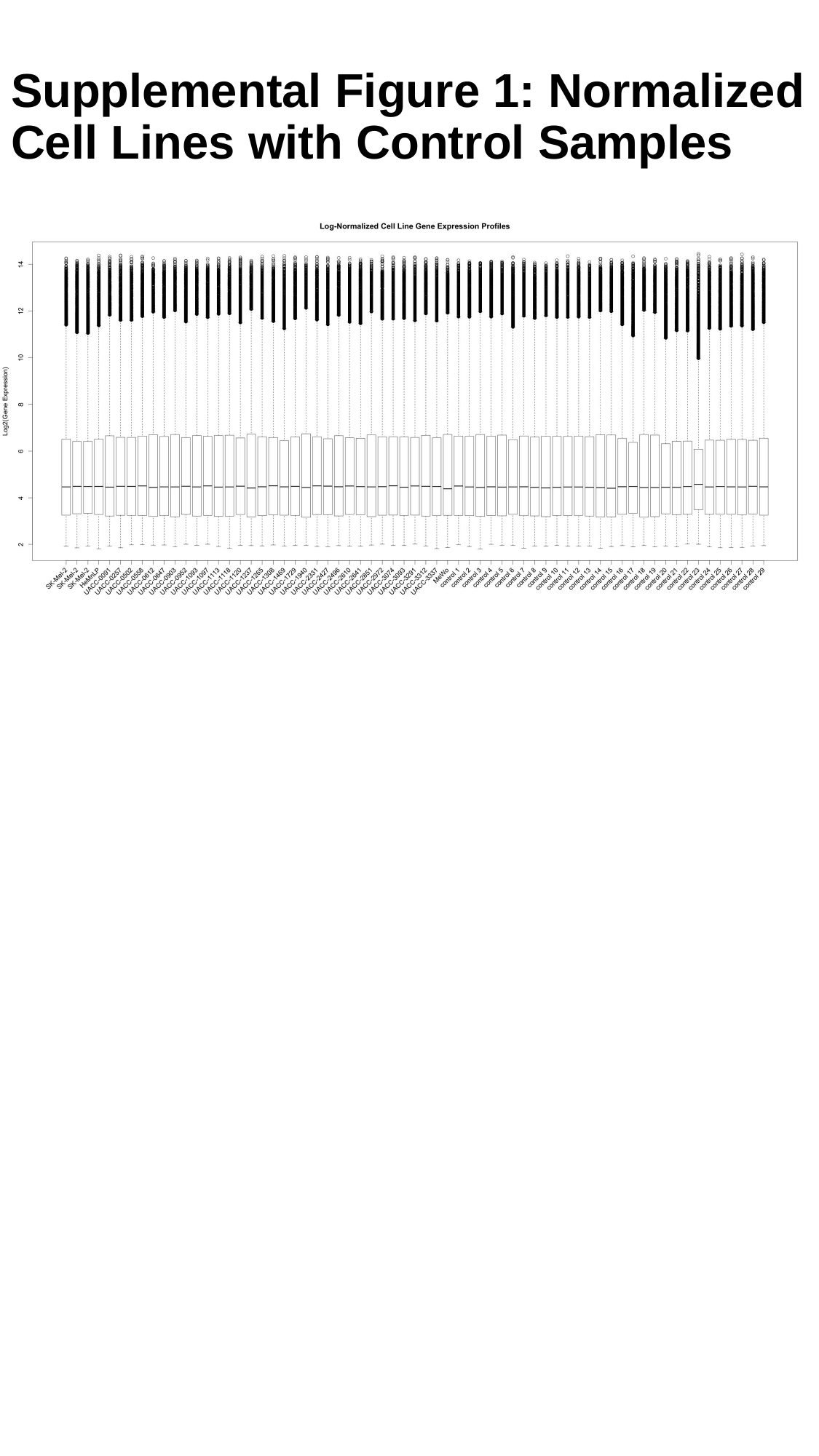

# Supplemental Figure 1: Normalized Cell Lines with Control Samples

## Slide 2
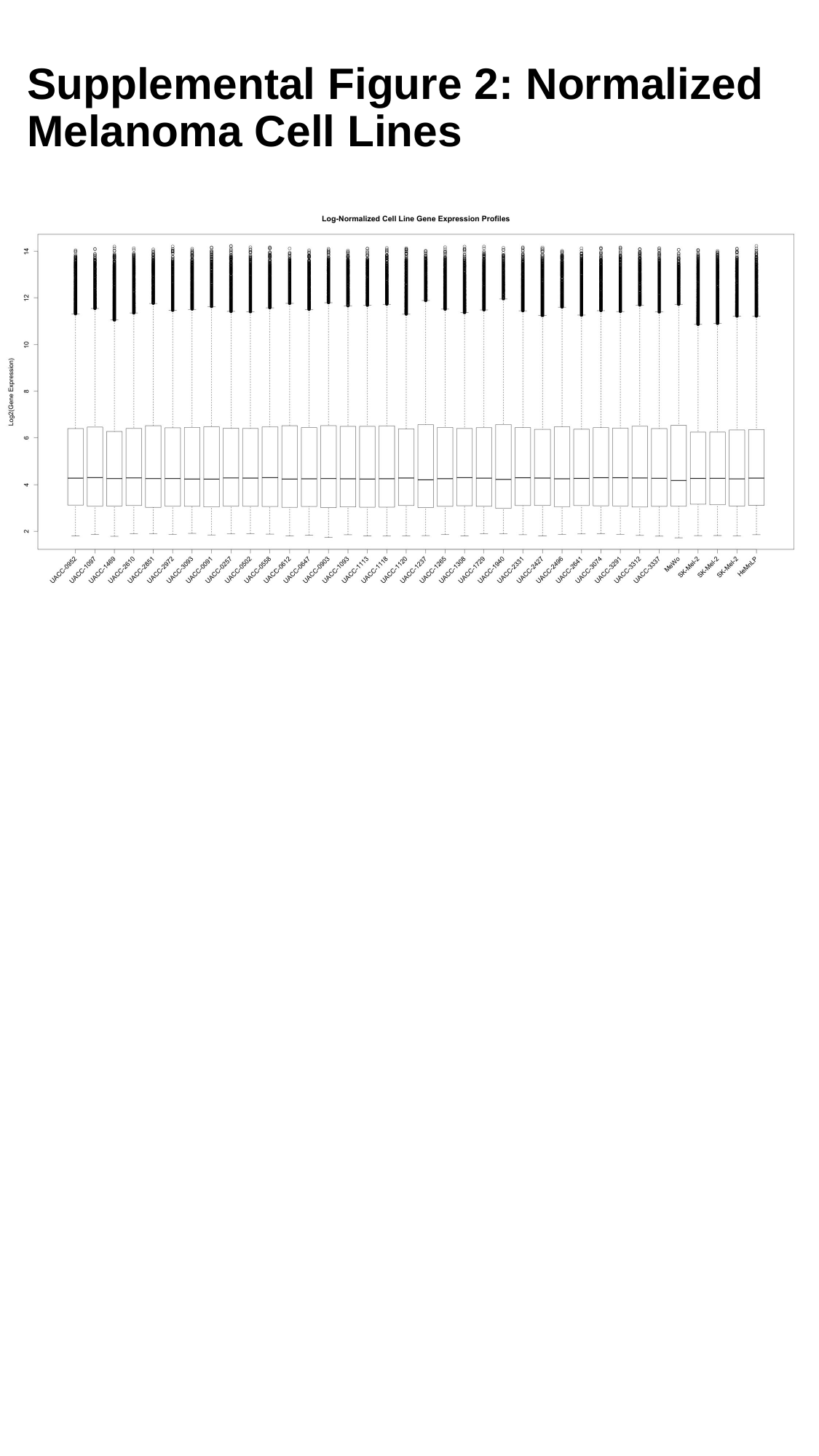

Supplemental Figure 2: Normalized Melanoma Cell Lines

## Slide 3
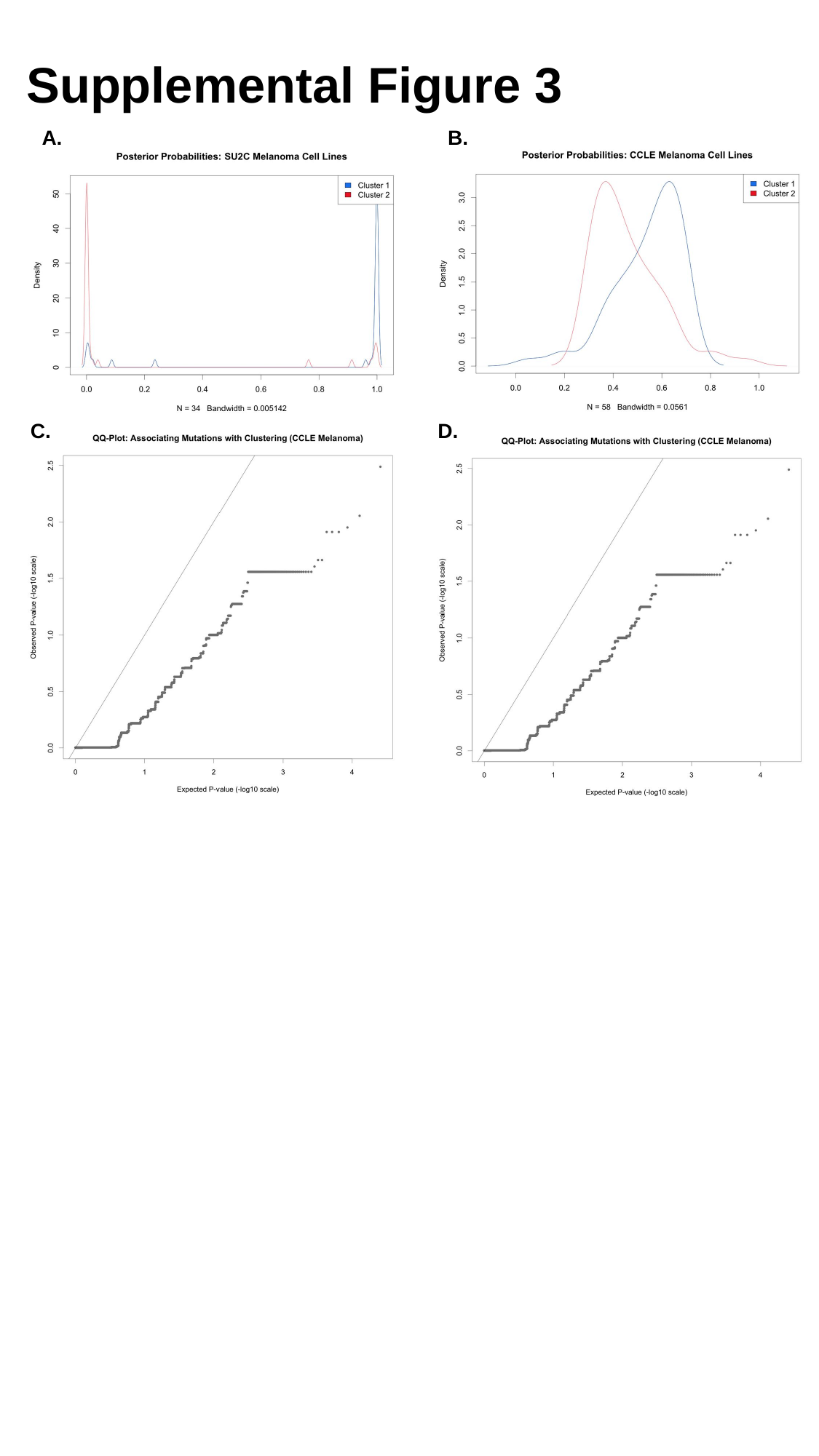

Supplemental Figure 3
A.
B.
C.
D.

## Slide 4
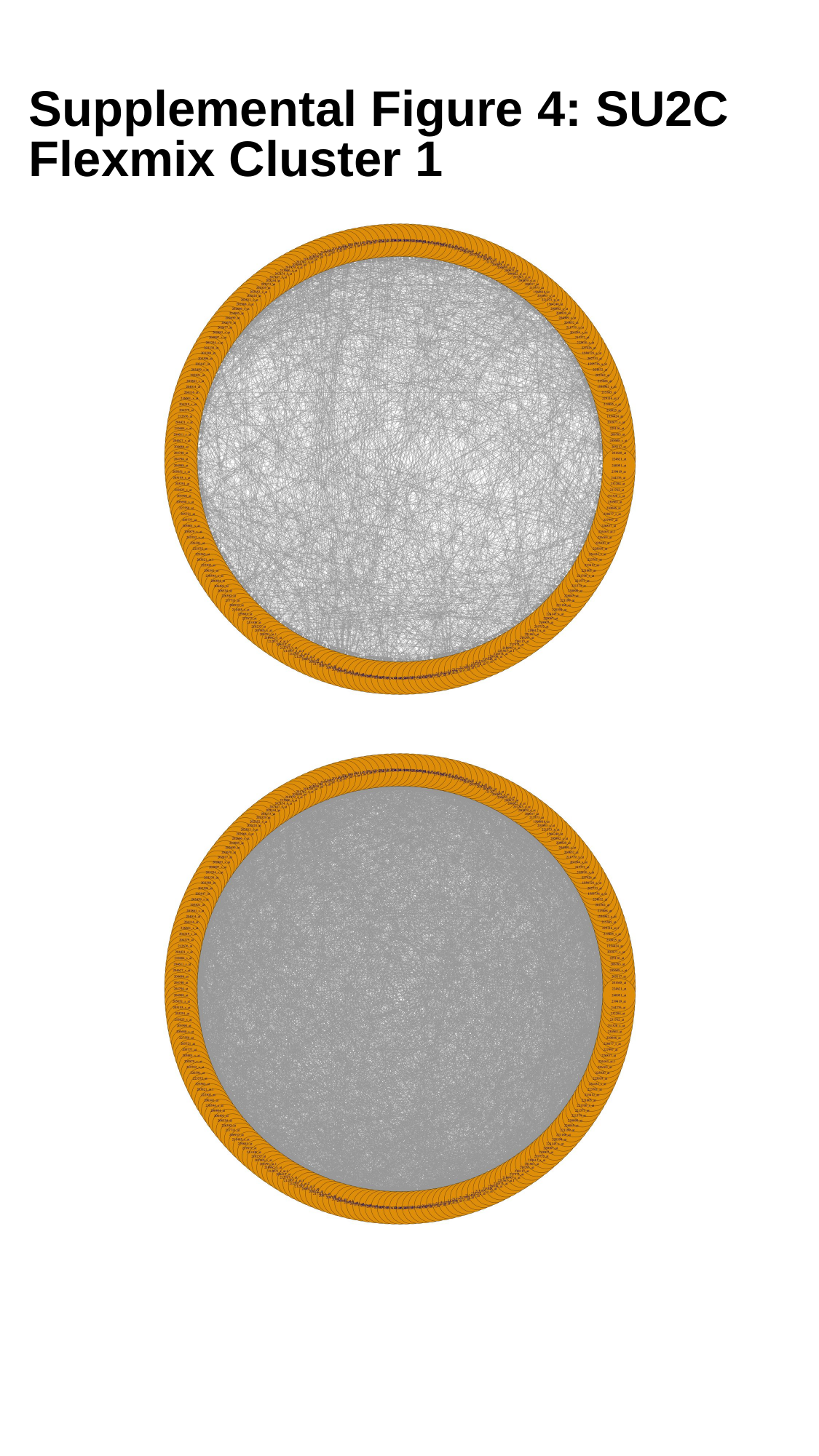

Supplemental Figure 4: SU2C Flexmix Cluster 1

## Slide 5
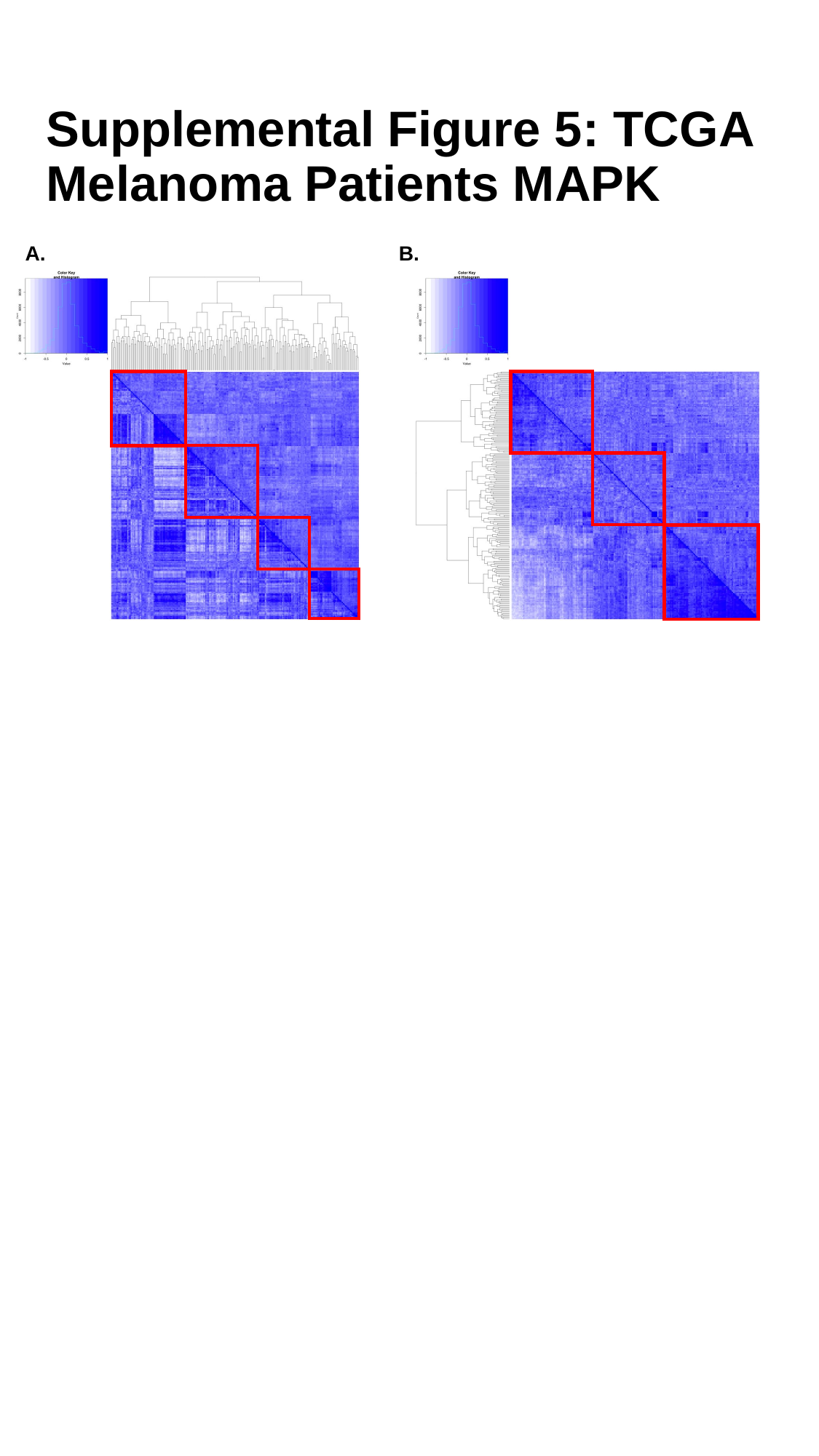

# Supplemental Figure 5: TCGA Melanoma Patients MAPK
A.
B.

## Slide 6
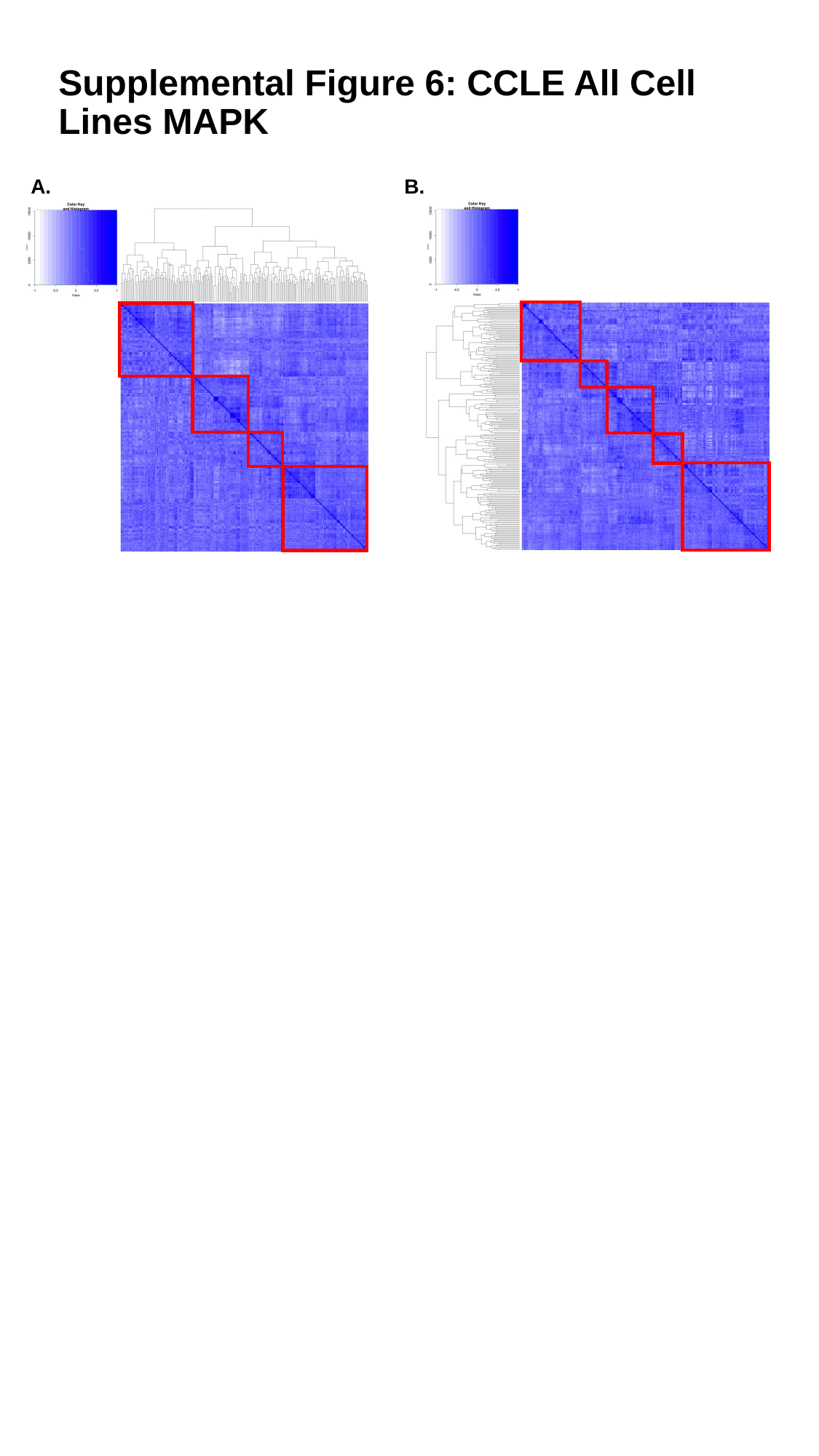

# Supplemental Figure 6: CCLE All Cell Lines MAPK
A.
B.

## Slide 7
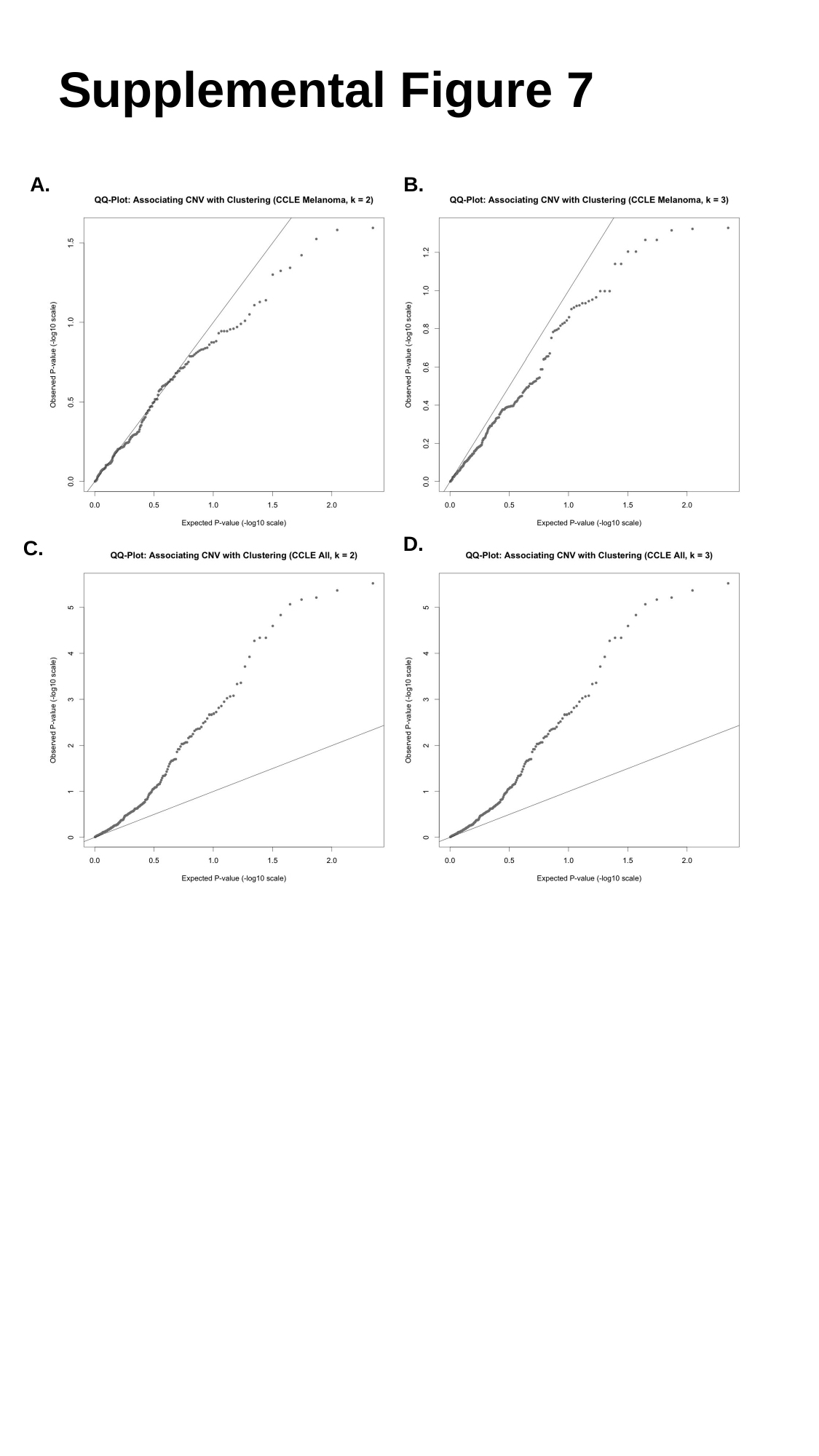

Supplemental Figure 7
A.
B.
D.
C.

## Slide 8
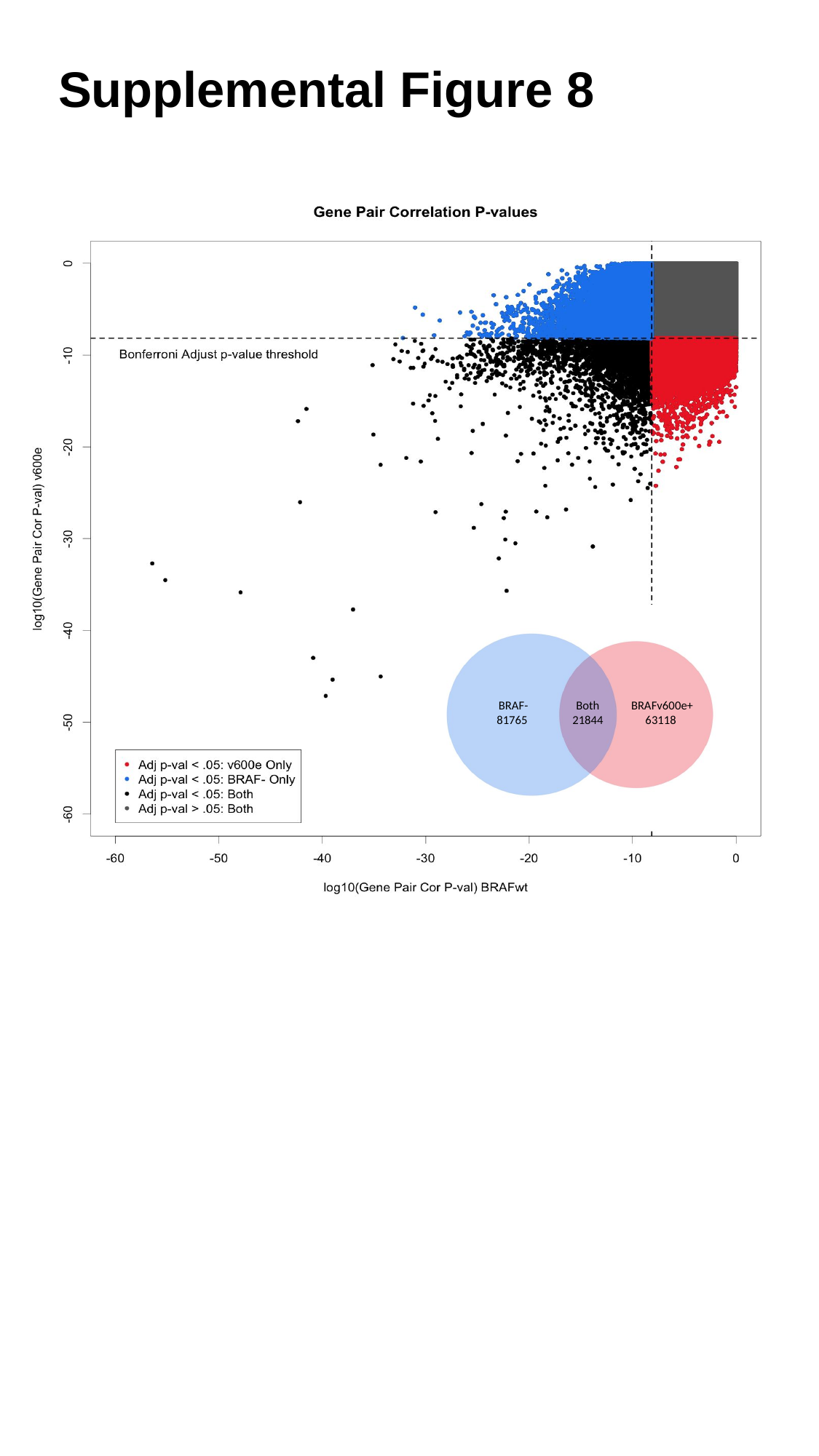

Supplemental Figure 8
BRAF-
81765
Both
21844
BRAFv600e+
63118

## Slide 9
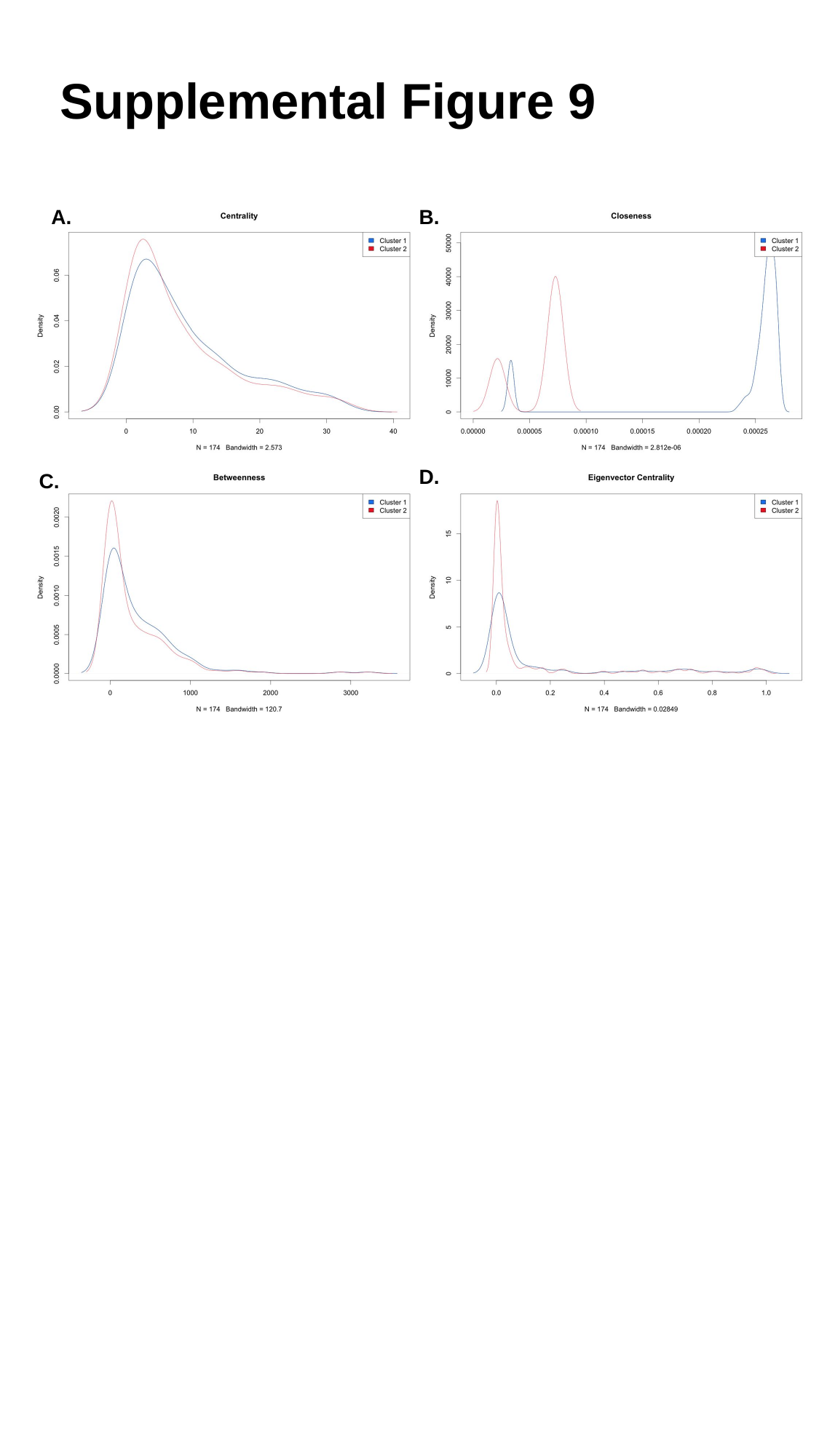

# Supplemental Figure 9
A.
B.
D.
C.

## Slide 10
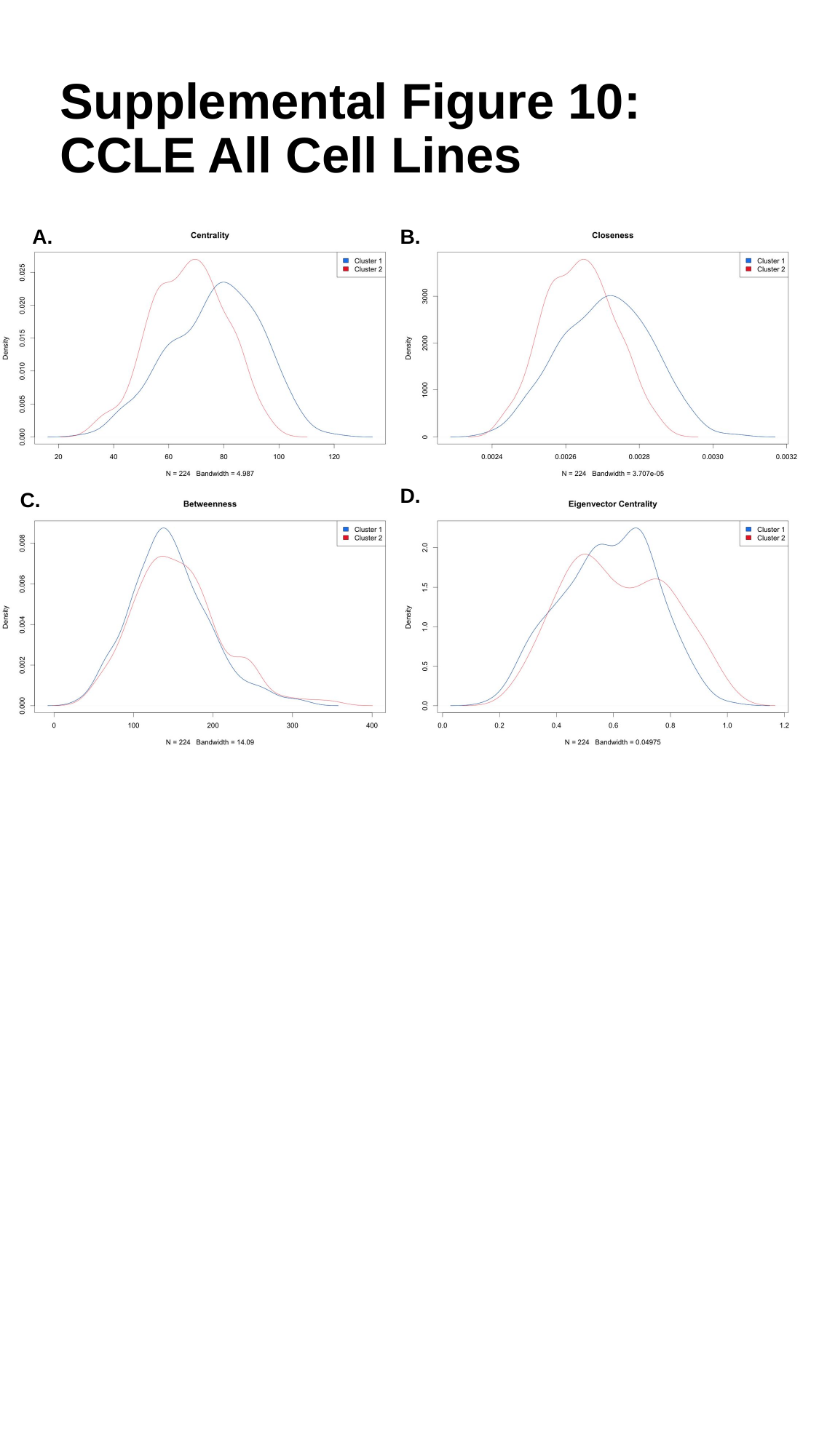

# Supplemental Figure 10: CCLE All Cell Lines
A.
B.
D.
C.

## Slide 11
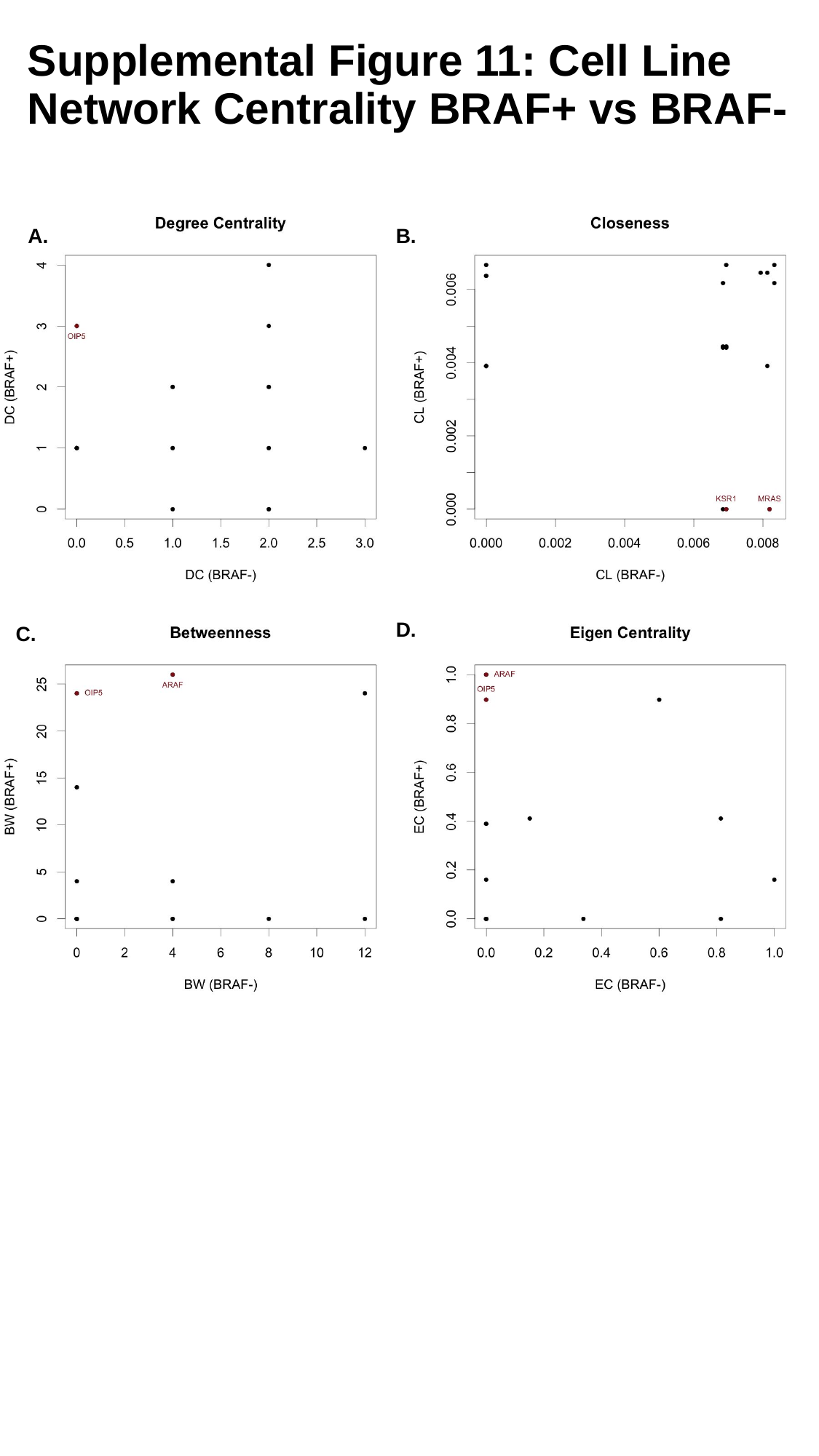

Supplemental Figure 11: Cell Line Network Centrality BRAF+ vs BRAF-
A.
B.
D.
C.

## Slide 12
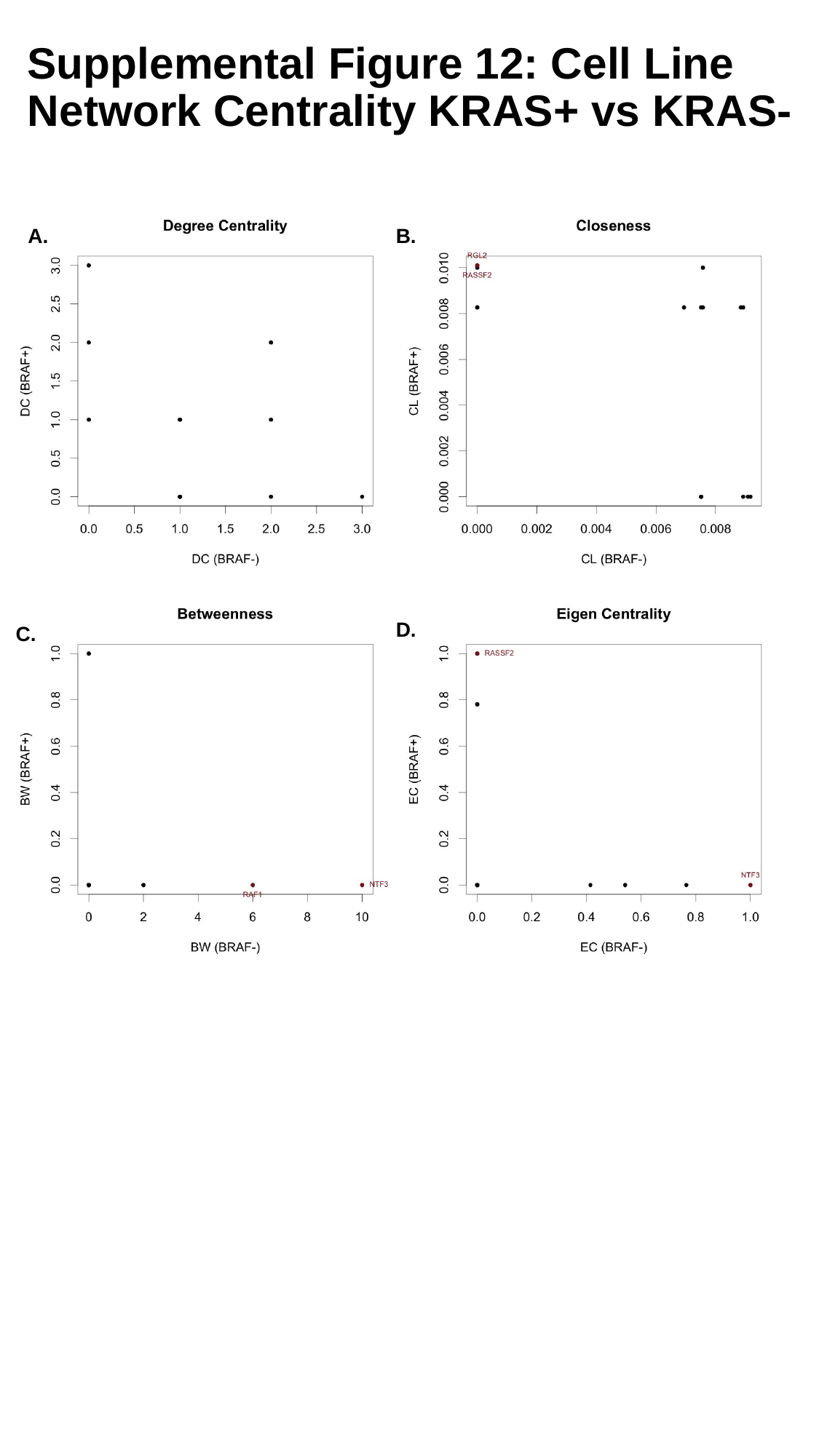

Supplemental Figure 12: Cell Line Network Centrality KRAS+ vs KRAS-
A.
B.
D.
C.

## Slide 13
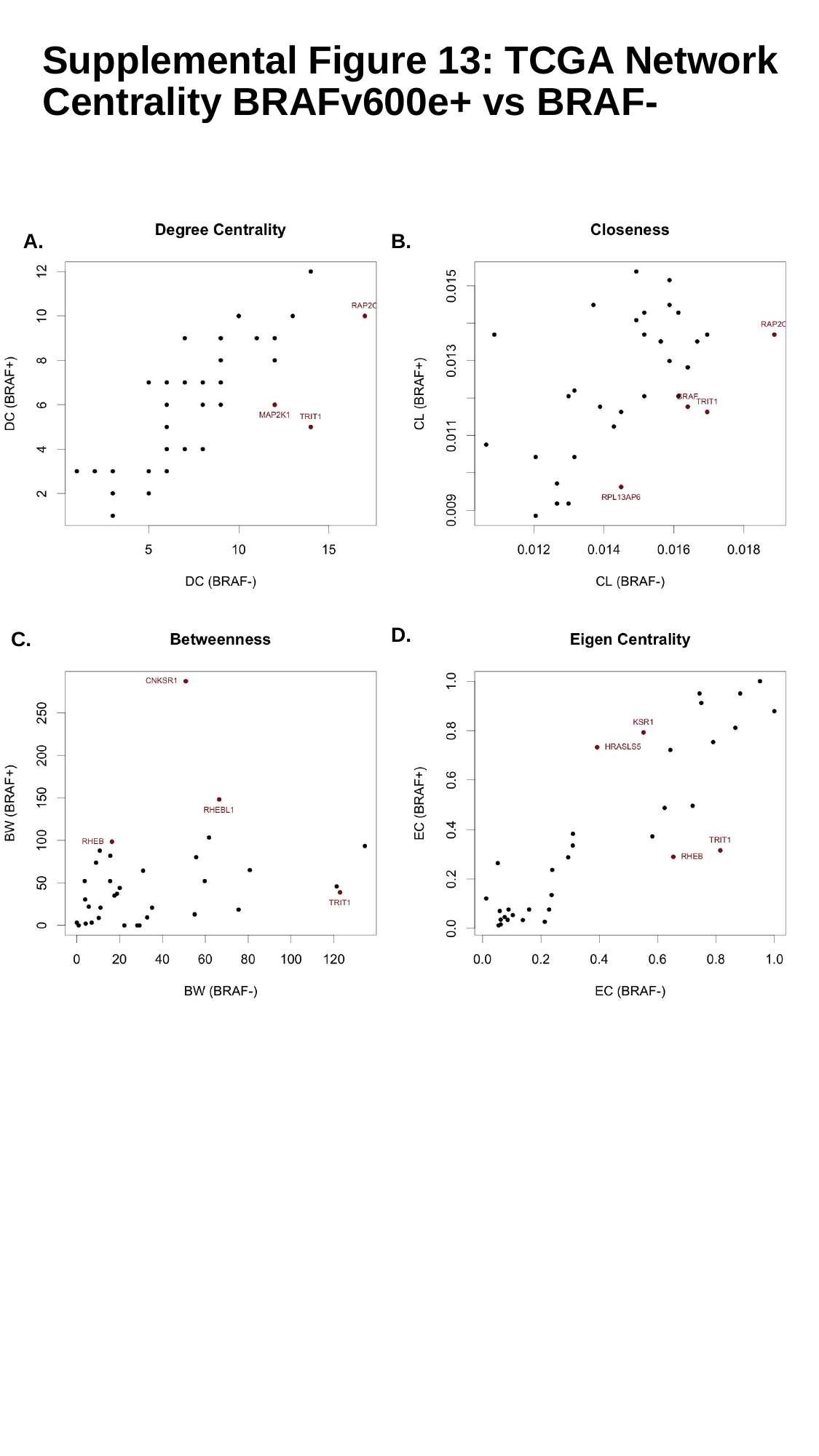

Supplemental Figure 13: TCGA Network Centrality BRAFv600e+ vs BRAF-
A.
B.
D.
C.

## Slide 14
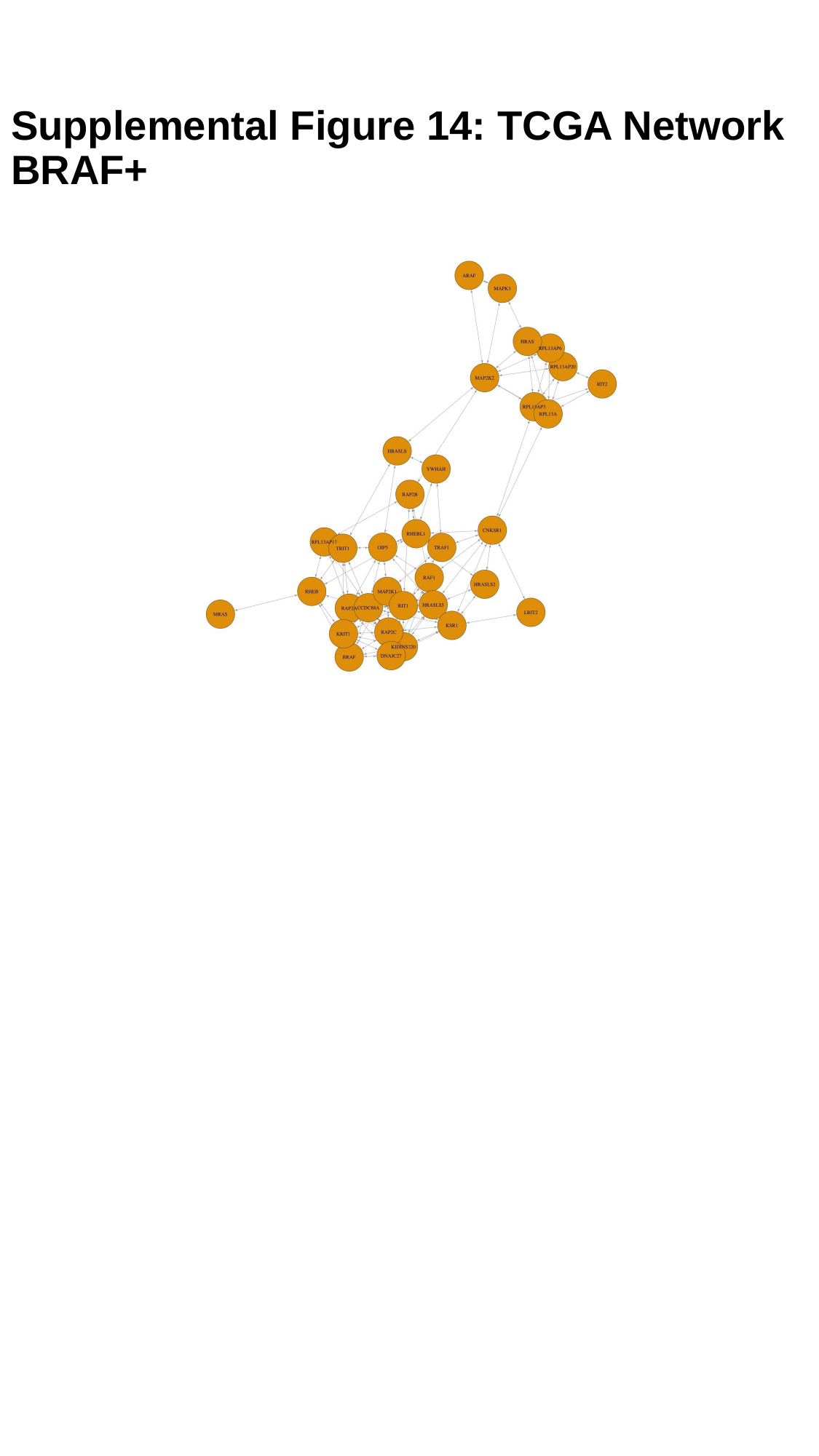

# Supplemental Figure 14: TCGA Network BRAF+

## Slide 15
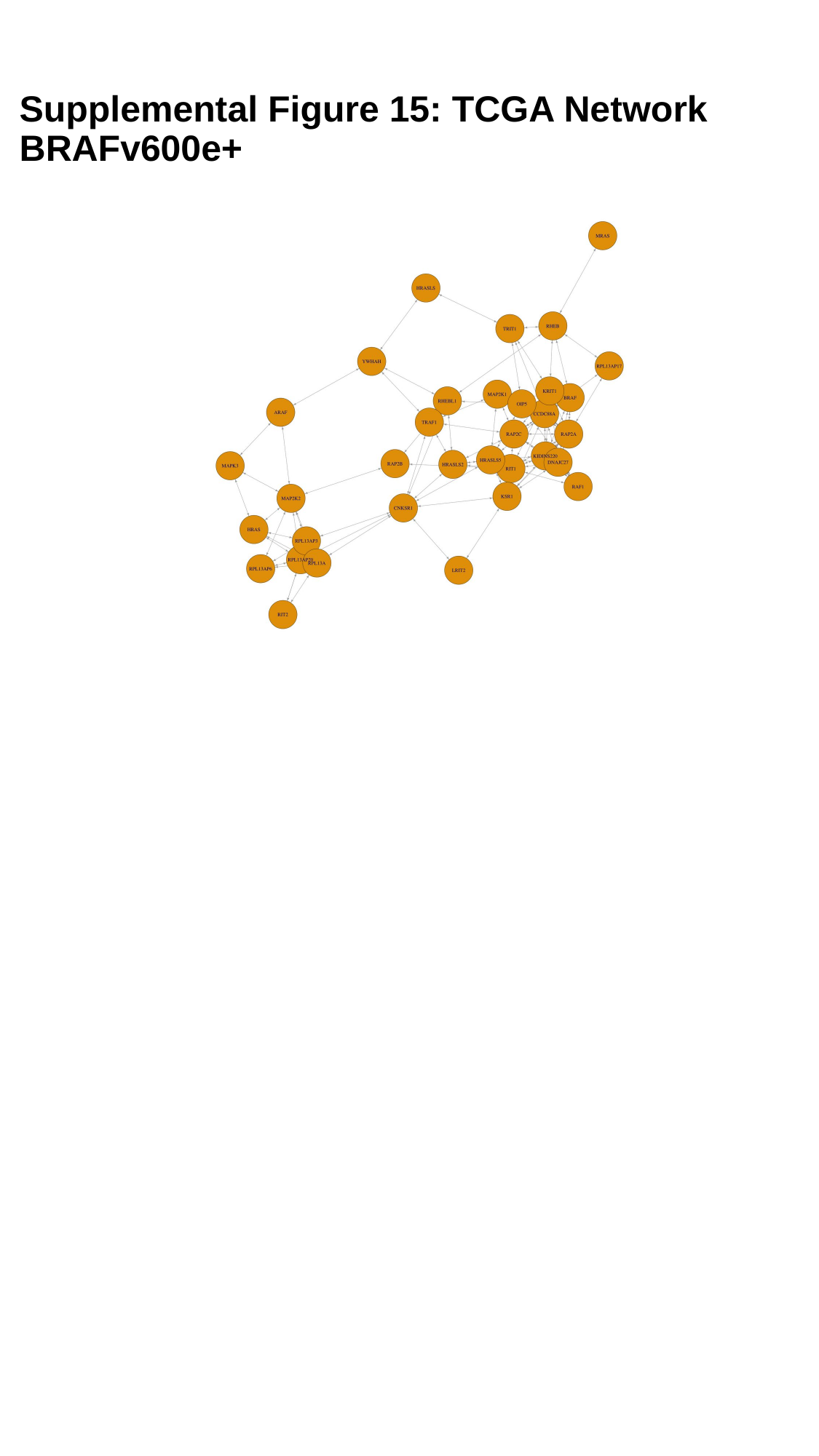

# Supplemental Figure 15: TCGA Network BRAFv600e+

## Slide 16
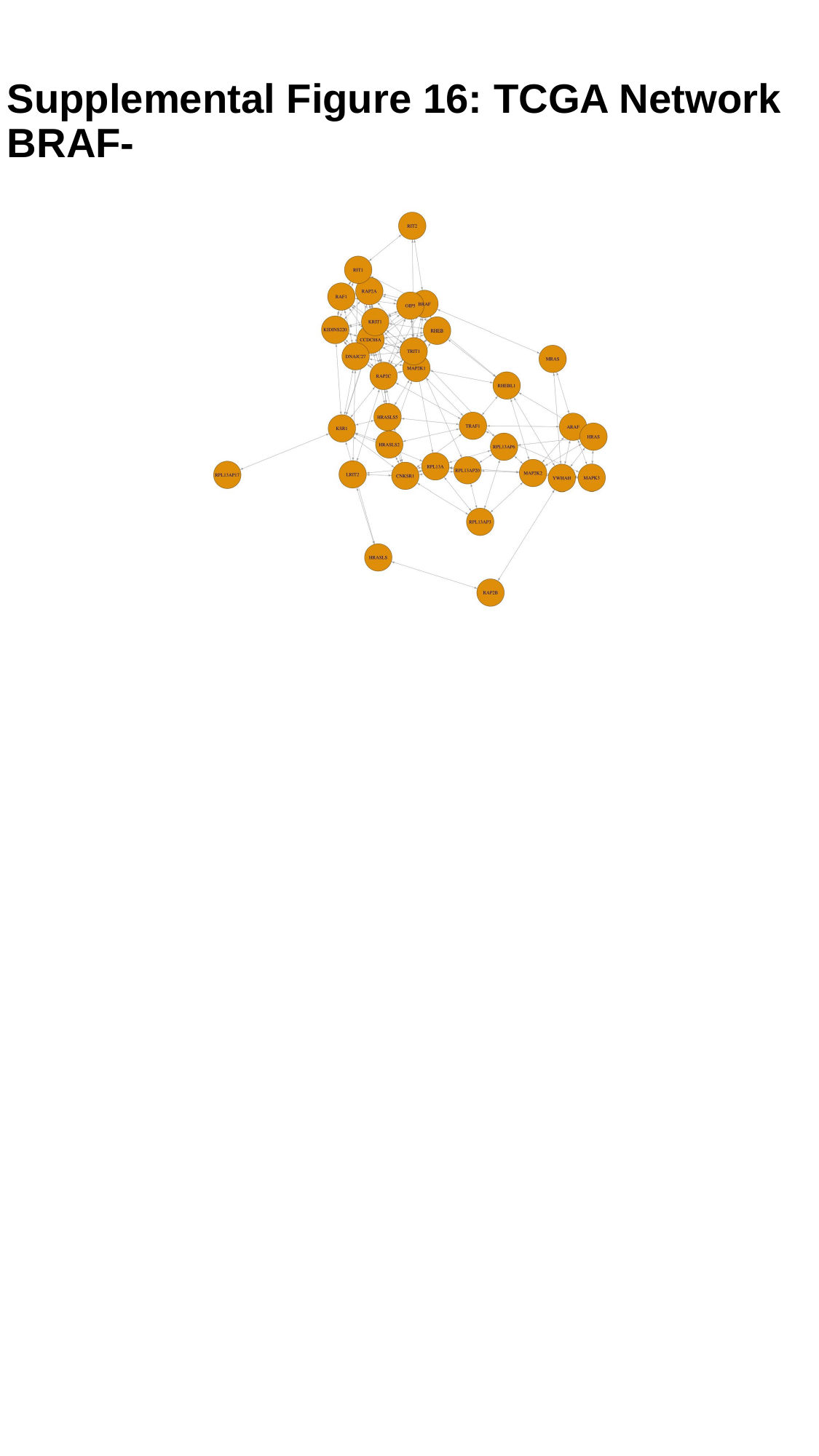

# Supplemental Figure 16: TCGA Network BRAF-

## Slide 17
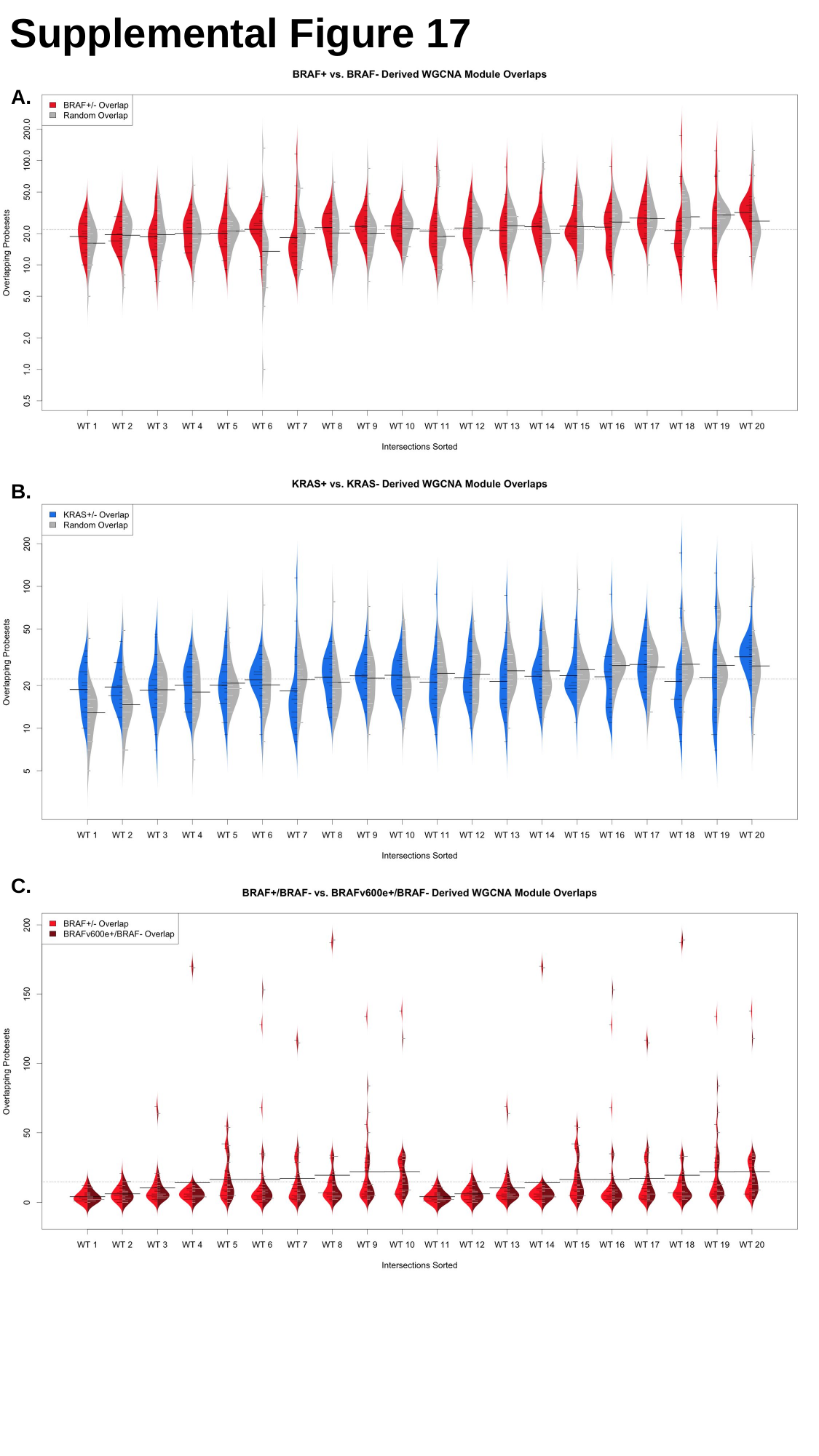

Supplemental Figure 17
A.
B.
C.

## Slide 18
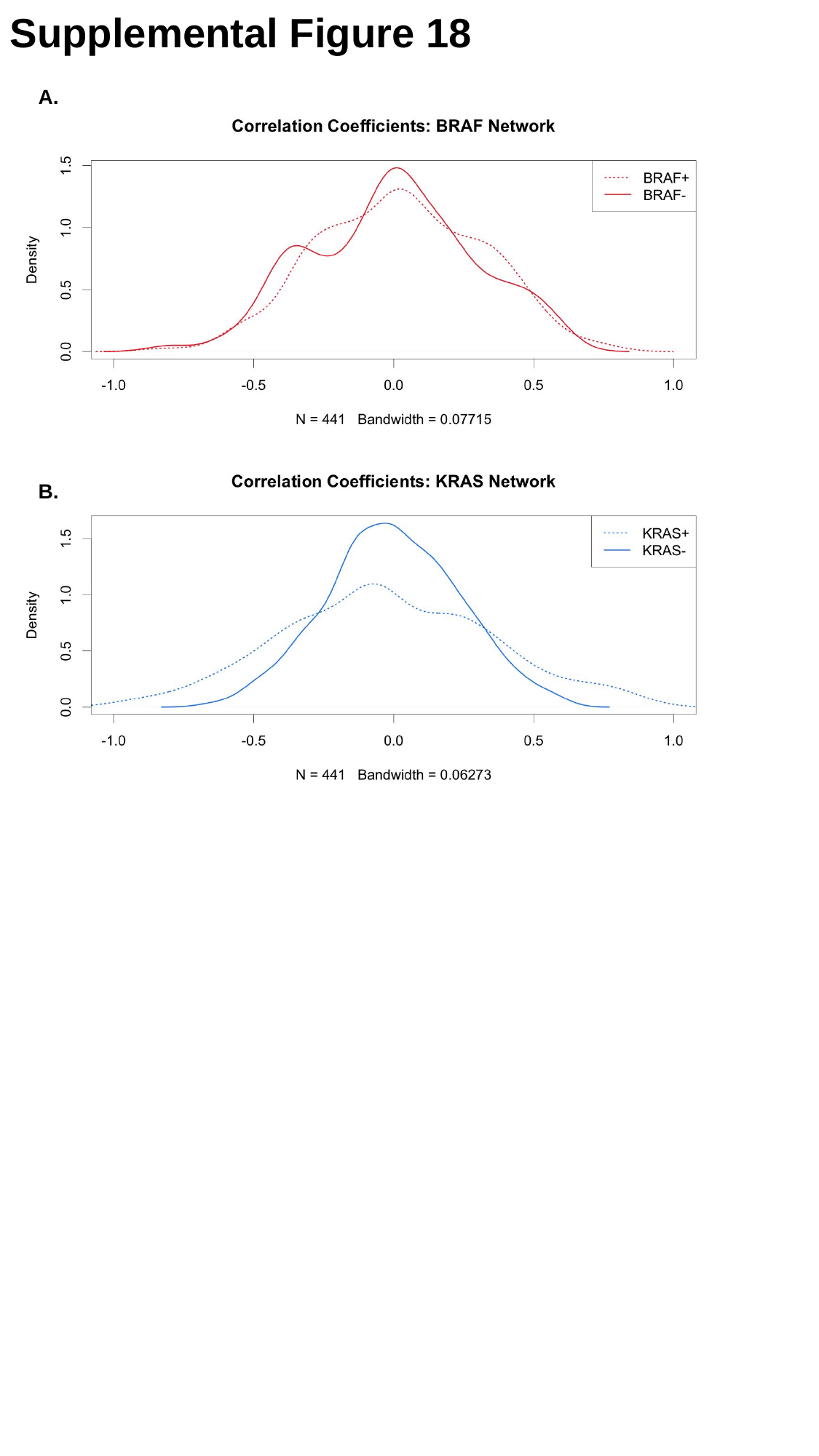

Supplemental Figure 18
A.
B.

## Slide 19
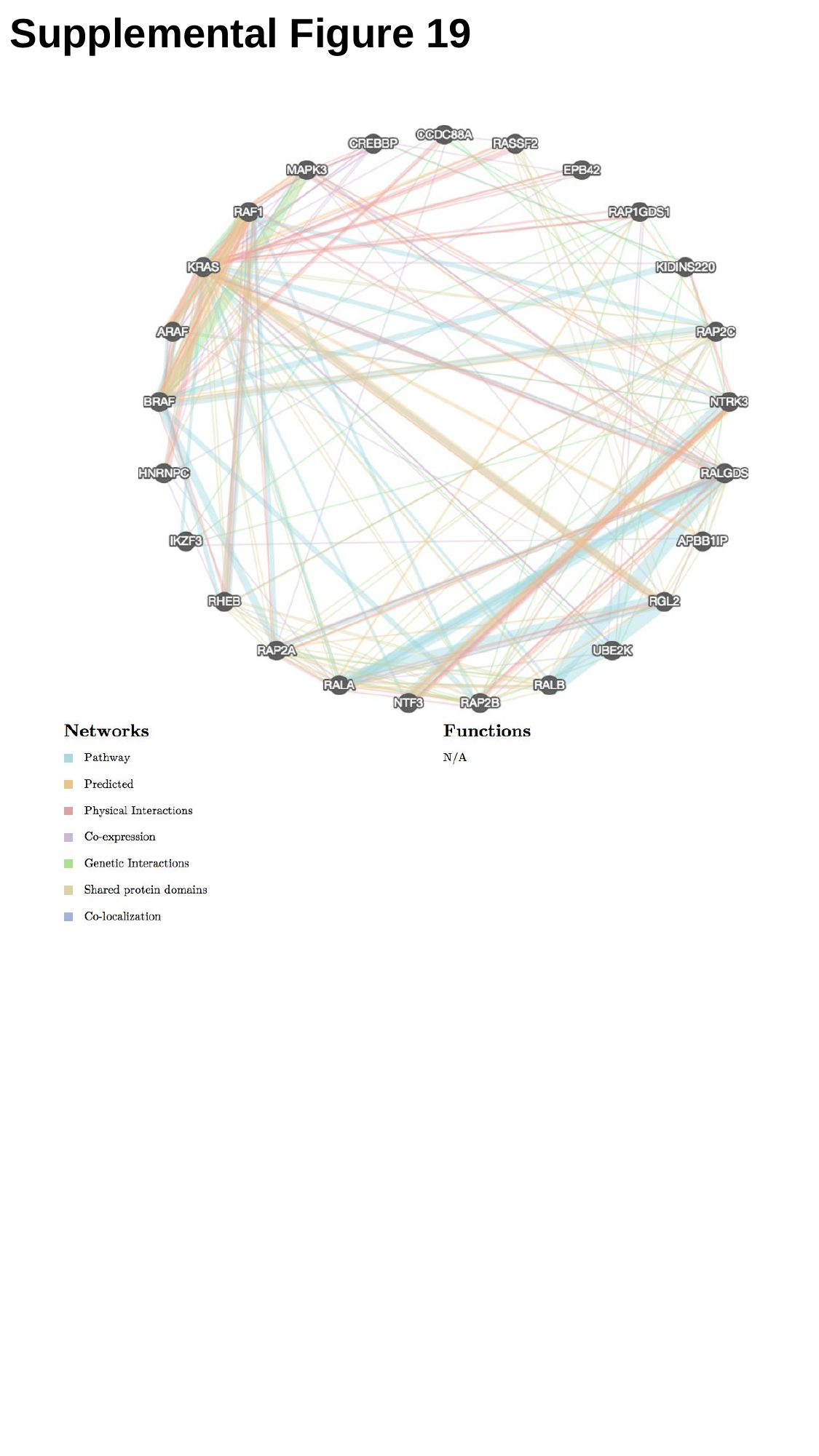

Supplemental Figure 19
